# Cryptotanshinone is a candidate therapeutic agent for interstitial lung disease associated with a BRICHOS-domain mutation of *SFTPC*

**DOI:** 10.1016/j.isci.2023.107731

**Published:** 2023-08-25

**Authors:** Motoyasu Hosokawa, Ryuta Mikawa, Atsuko Hagiwara, Yukiko Okuno, Tomonari Awaya, Yuki Yamamoto, Senye Takahashi, Haruka Yamaki, Mitsujiro Osawa, Yasuhiro Setoguchi, Megumu K. Saito, Shinji Abe, Toyohiro Hirai, Shimpei Gotoh, Masatoshi Hagiwara

**Affiliations:** 1 Department of Anatomy and Developmental Biology, Graduate School of Medicine, Kyoto University, Sakyo-ku, Kyoto 606-8501, Japan; 2 Department of Developmental Biology and Functional Genomics, Ehime University Graduate School of Medicine, Toon, Ehime 791-0295, Japan; 3 Department of Respiratory Medicine, Graduate School of Medicine, Kyoto University, Kyoto 606-8507, Japan; 4 Department of Drug Discovery for Lung Diseases, Graduate School of Medicine, Kyoto University, Kyoto 606-8501, Japan; 5 Department of Clinical Application, Center for iPS Cell Research and Application (CiRA), Kyoto University, Kyoto 606-8507, Japan; 6 Medical Research Support Center, Graduate School of Medicine, Kyoto University, Sakyo-ku, Kyoto 606-8501, Japan; 7 Department of Respiratory Medicine, Graduate School of Medical and Dental Sciences, Tokyo Medical and Dental University (TMDU), Tokyo 113-8519, Japan; 8 Department of Respiratory Medicine Tokyo, Medical University Hospital, Shinjuku-ku, Tokyo 160-0023, Japan

**Keywords:** Biochemistry, Molecular biology, Cell biology, Stem cells research

## Abstract

Interstitial lung disease (ILD) represents a large group of diseases characterized by chronic inflammation and fibrosis of the lungs, for which therapeutic options are limited. Among several causative genes of familial ILD with autosomal dominant inheritance, the mutations in the BRICHOS domain of *SFTPC* cause protein accumulation and endoplasmic reticulum stress by misfolding its proprotein. Through a screening system using these two phenotypes in HEK293 cells and evaluation using alveolar epithelial type 2 (AT2) cells differentiated from patient-derived induced pluripotent stem cells (iPSCs), we identified Cryptotanshinone (CPT) as a potential therapeutic agent for ILD. CPT decreased cell death induced by mutant SFTPC overexpression in A549 and HEK293 cells and ameliorated the bleomycin-induced contraction of the matrix in fibroblast-dependent alveolar organoids derived from iPSCs with *SFTPC* mutation. CPT and this screening strategy can apply to abnormal protein-folding-associated ILD and other protein-misfolding diseases.

## Highlights

• A phenotypic screening system targeting *SFTPC* mutation was established
• ILD patient-derived iPSC were applied to evaluating candidate chemical compounds
• Cryptotanshinone suppressed cell death caused by *SFTPC* mutation
• Cryptotanshinone ameliorated the fibrosis phenotype of the patient-derived iPSC

## Graphical abstract

**Figure:**
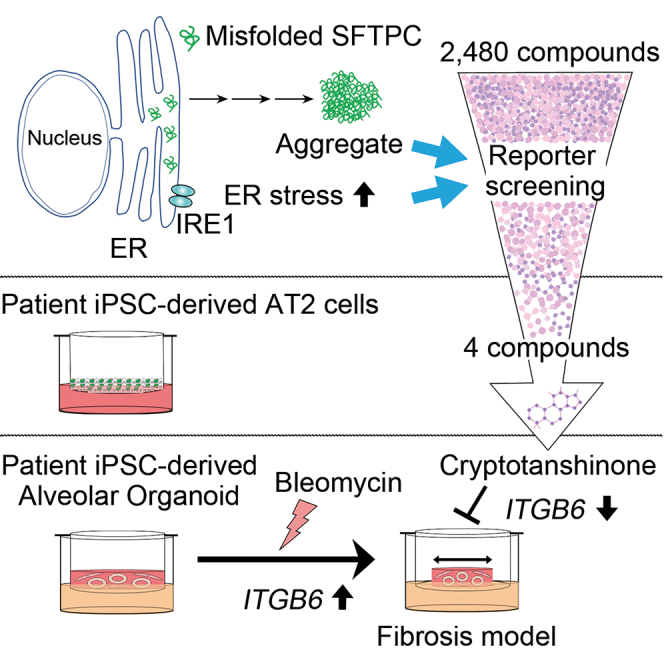

## Introduction

Interstitial lung disease (ILD) comprises a large group of diseases that cause lung fibrosis. Patients with progressive fibrosing ILD (PF-ILD) have poor prognoses and often experience severe respiratory failure. Its mechanism has not yet been elucidated; however, some genetic mutations associated with alveolar type 2 (AT2) cells have been reported to cause progressive pulmonary fibrosis, making it difficult to breathe and supply oxygen to the bloodstream.^1^^,^^2^

In recent years, nintedanib and pirfenidone were approved by the US Food and Drug Administration (FDA) for treating progressive pulmonary fibrosis, although they cannot halt and reverse fibrosis that has already occurred.^3^^,^^4^ The pathogenesis of pulmonary fibrosis is initiated by alveolar epithelial injury, followed by fibroblast activation. Therefore, there is an increasing demand for new therapeutic drugs targeting the alveolar epithelial cells involved in the early stages of ILD. The causes of initial alveolar epithelial injury are diverse, including the side effects of drugs such as amiodarone and environmental factors such as cigarette smoke.^5^ Genetic mutations have also been found to be associated with ILD. Recently, the development of next-generation sequencing has suggested that more than ten genes are responsible for familial ILD. In particular, many reports have indicated that mutations in genes critical for surfactant metabolism and functions [*surfactant protein C* (*SFTPC*), *surfactant protein A1/A2* (*SFTPA1/A2*), and *ATP binding cassette subfamily A member 3* (*ABCA3*)] are associated with ILD.^6^ Among them, more than 70 *SFTPC* mutations associated with ILD have been described and found in a large and heterogeneous group of patients with sporadic and familial ILD with autosomal dominant inheritance.^7^^,^^8^ Notably, many of these mutations occur in the distal C-terminal (residues 94–197) of the BRICHOS domain of *SFTPC*^9^ and cause misfolding, which results in unfolded protein responses such as Endoplasmic Reticulum (ER) stress, and leads to aberrant trafficking, cytosolic aggregation, and aggresome formation.^10^^,^^11^^,^^12^^,^^13^^,^^14^^,^^15^ This study established a compound screening system utilizing two molecular phenotypes, ER stress, and SFTPC aggregates; both are involved in the disease-causing mechanisms invoked by BRICHOS domain mutations. Furthermore, since our group previously established methods for generating human induced pluripotent stem cells (iPSC)-derived alveolar cells in organoids,^16^^,^^17^ AT2 cells differentiated from patient-specific iPSC harboring *SFTPC* mutations that cause ILD were used to evaluate the efficacy of the candidate compounds, and we identified Cryptotanshinone (CPT) as a potential therapeutic agent for ILD.

## Results

### High-throughput screening and identification of compounds that reduce ER stress caused by mutant *SFTPC*

**Figure 1:**
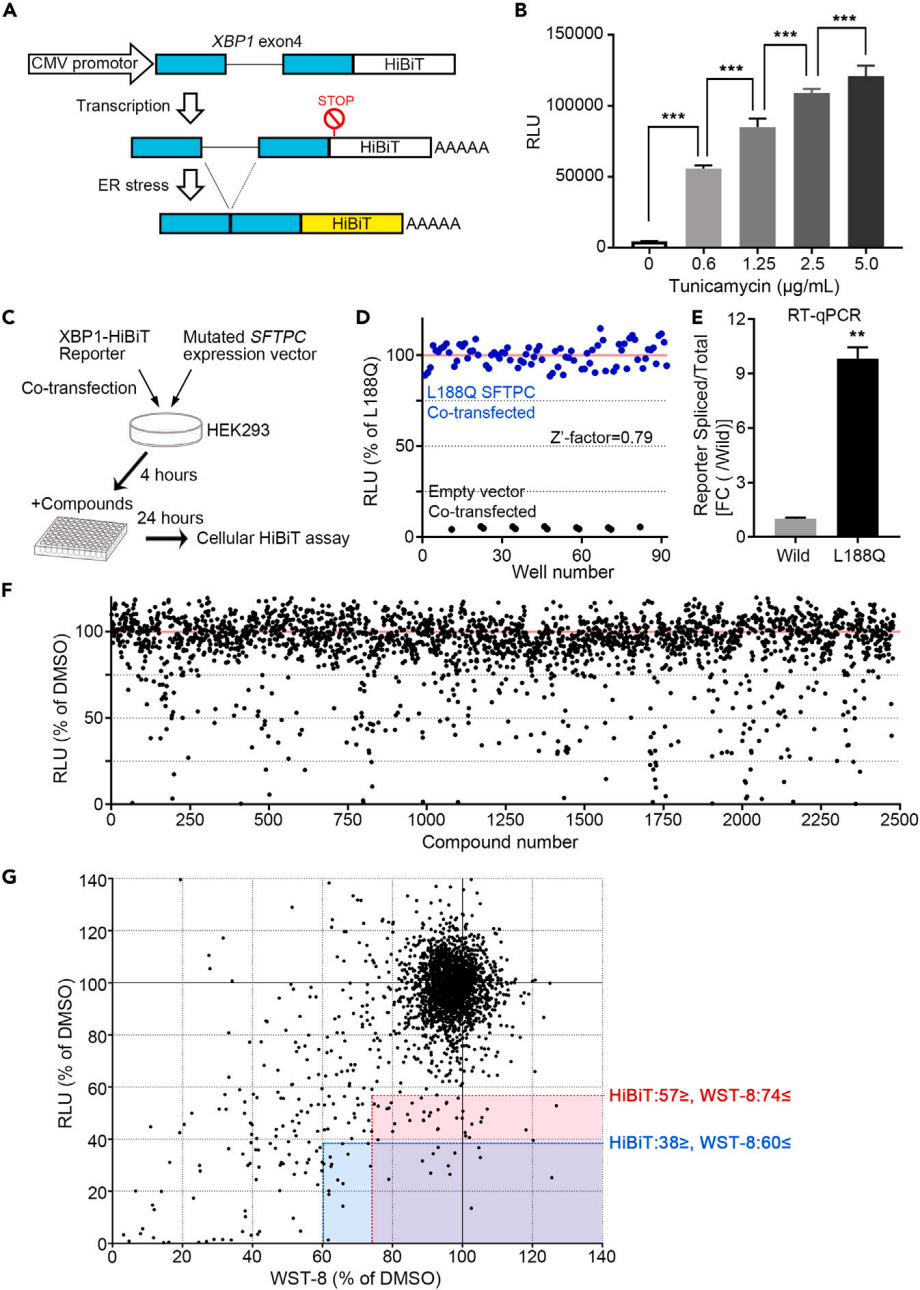
High-throughput screening and identification of compounds that reduce ER stress caused by mutant *SFTPC* (A) Diagram illustrating the construction of XBP1-HiBiT Reporter. It was spliced under ER stress and resulted in the translation of a spliced XBP1(exon 4)-HiBiT fusion protein. The STOP sign indicates the stop codon of the open reading frame of unspliced mRNA. (B) Cellular HiBiT assay using the Nano-Glo HiBiT Lytic Detection System for HEK293 cells transfected with XBP1-HiBiT Reporter and treated with tunicamycin (0.6, 1.25, 2.5 or 5.0 μg/mL) or DMSO (0.05%) for 6 h (n = 6). Data are presented as mean ± SD. ∗∗∗p < 0.001 [One-way analysis of variant (ANOVA) with Tukey’s multiple comparisons test]. (C) Diagram illustrating the screening method. (D) Validation of high-throughput screening using the cellular HiBiT assay. HEK293 cells were co-transfected with XBP1-HiBiT Reporter and empty or L188Q-mutant SFTPC expression vector (L188Q SFTPC). n = 80 (L188Q SFTPC), n = 12 (empty vector). (E) RT-qPCR of the splicing ratio of XBP1-HiBiT Reporter (spliced/total expression) in HEK293 cells subjected to 24 h incubation with co-transfected XBP1-HiBiT Reporter and wild or L188Q SFTPC expression vectors. Data are presented as mean ± SD (n = 3 independent experiments). ∗∗p < 0.01 (Welch’s t test). (F) Scatterplot for relative luminescence units (RLU) (% of DMSO) analyzed by XBP1-HiBiT Reporter co-transfected with L188Q SFTPC expression vector in the screening of 2480 compounds. Data are presented as mean (n = 2). (G) Scatterplot showing pairwise comparisons of the result of WST-8 assay (n = 2) (% of DMSO) versus the result of HiBiT assay (from Figure 1F) in the screening of the 2480 compounds. Red and blue characters and lines indicate each threshold to identify hit compounds. See also Figure S1.

First, we focused on ER stress among the molecular pathogeneses caused by *SFTPC* with BRICHOS domain mutations and developed a high-throughput screening system. To detect ER stress, we used an *X-box binding protein 1* (*XBP1*) splicing reporter, which was constructed by fusing HiBiT protein, the split particle of NanoLuc luciferase,^18^ to exon 4 of the *XBP1* containing inositol requiring enzyme 1 (IRE1)-mediated splice sites (hereinafter called “XBP1-HiBiT Reporter”) (Figure 1A). After an unfolded protein response induces ER stress, the sensor protein IRE1 is activated. Activated IRE1 mediates the splicing of 26 nucleotides of exon 4 of *XBP1* in the cytoplasm, and the spliced XBP1 functions as a nuclear transcription factor. This *XBP1* splicing system has been used to indicate ER stress.^19^^,^^20^ We examined whether the XBP1-HiBiT Reporter could quantitatively detect ER stress using tunicamycin, commonly used to induce ER stress. In HEK293 cells transfected with the XBP1-HiBiT Reporter, the relative light unit (RLU) increased in a concentration-dependent manner with tunicamycin (Figure 1B), indicating that the XBP1-HiBiT Reporter could be used to evaluate ER stress quantitatively. Next, we constructed an expression vector of myc-tagged *SFTPC* with the L188Q mutation (L188Q SFTPC) and the wild-type *SFTPC* (wild SFTPC). *SFTPC* encodes L188Q mutation with a mutation (c.563T>A) and substitutes glutamine for leucine at amino acid 188 in the BRICHOS domain of the *SFTPC*. The L188Q mutation in *SFTPC* was reported as a causative mutation in autosomal dominant familial ILD and an ER stress inducer.^13^^,^^21^ By co-transfection of L188Q SFTPC with the XBP1-HiBiT Reporter, we generated a system that could evaluate the effect of compounds on ER stress caused by the BRICHOS *SFTPC* mutant (Figure 1C). The Z′-factor, an indicator of the statistical confidence of screening, was 0.79 for this screening assay, which is considered high enough for screening^22^ (Figure 1D). To confirm that L188Q SFTPC induces ER stress in this system, we compared the splicing ratio of the XBP1-HiBiT Reporter using reverse transcription-quantitative polymerase chain reaction (RT-qPCR) analysis. The splicing ratio was significantly higher in HEK293 cells co-transfected with L188Q SFTPC and the reporter than those co-transfected with wild SFTPC and the reporter (Figure 1E). We further analyzed the changes in protein and mRNA expression levels of ER stress marker genes. In RT-qPCR analysis, mRNAs levels of *HSPA5* [*heat shock protein family A (Hsp70) member 5*] (*BIP*), *DDIT3* (*DNA damage-inducible transcript 3) (CHOP)* and *ATF4 (activating transcription factor 4*), and endogenous *XBP1* splicing ratio were significantly increased in L188Q SFTPC overexpressed cells relative to wild SFTPC overexpressed cells (Figure S1A). In western blotting (WB) analysis, BIP and CHOP protein levels were significantly increased in L188Q SFTPC overexpressed cells relative to wild SFTPC overexpressed cells (Figure S1B). To confirm the difference in transfection efficiency, we examined the expression level of neomycin phosphotransferase II (neomycin-resistance gene), which is expressed from a different promoter of the same plasmid (pcDNA3). There was no large difference in the expression levels between wild and L188Q SFTPC (Figure S1B), indicating transfection efficiency of wild and L188Q SFTPC expression vector is almost the same. These results suggest that L188Q SFTPC induces ER stress in this screening system.

We screened 2,480 compounds from the Kyoto University chemical library to identify compounds that could reduce ER stress caused by L188Q SFTPC overexpression (Figure 1F). As a positive control to reduce ER stress, we used 5 mM 4-phenyl butyric acid (4-PBA).^23^^,^^24^ To exclude highly toxic compounds, the cytotoxicity of the compounds at 10 μM was confirmed using a Water-Soluble Tetrazolium 8 (WST-8) assay, which measures intracellular dehydrogenase activity as an indicator of cell viability. Positive control (4-PBA): RLU of HiBiT = 51.1% ± 6.4 and WST-8 = 77.2% ± 3.3 [mean ± SD, % of dimethyl sulfoxide (DMSO)]. The threshold to determine the hit compounds was calculated based on HiBiT and WST-8 assay results for the positive control (4-PBA) because 4-PBA was reported to reduce ER stress caused by SFTPC BRICHOS mutant^23^ (HiBiT: 57% and WST-8: 74%). Another threshold was used to obtain highly effective compounds with some toxicity (HiBiT: 38% and WST-8: 60%). Sixty-five hit compounds were identified (Figure 1G).

### Secondary screening for compounds identified in the primary screening

**Figure 2:**
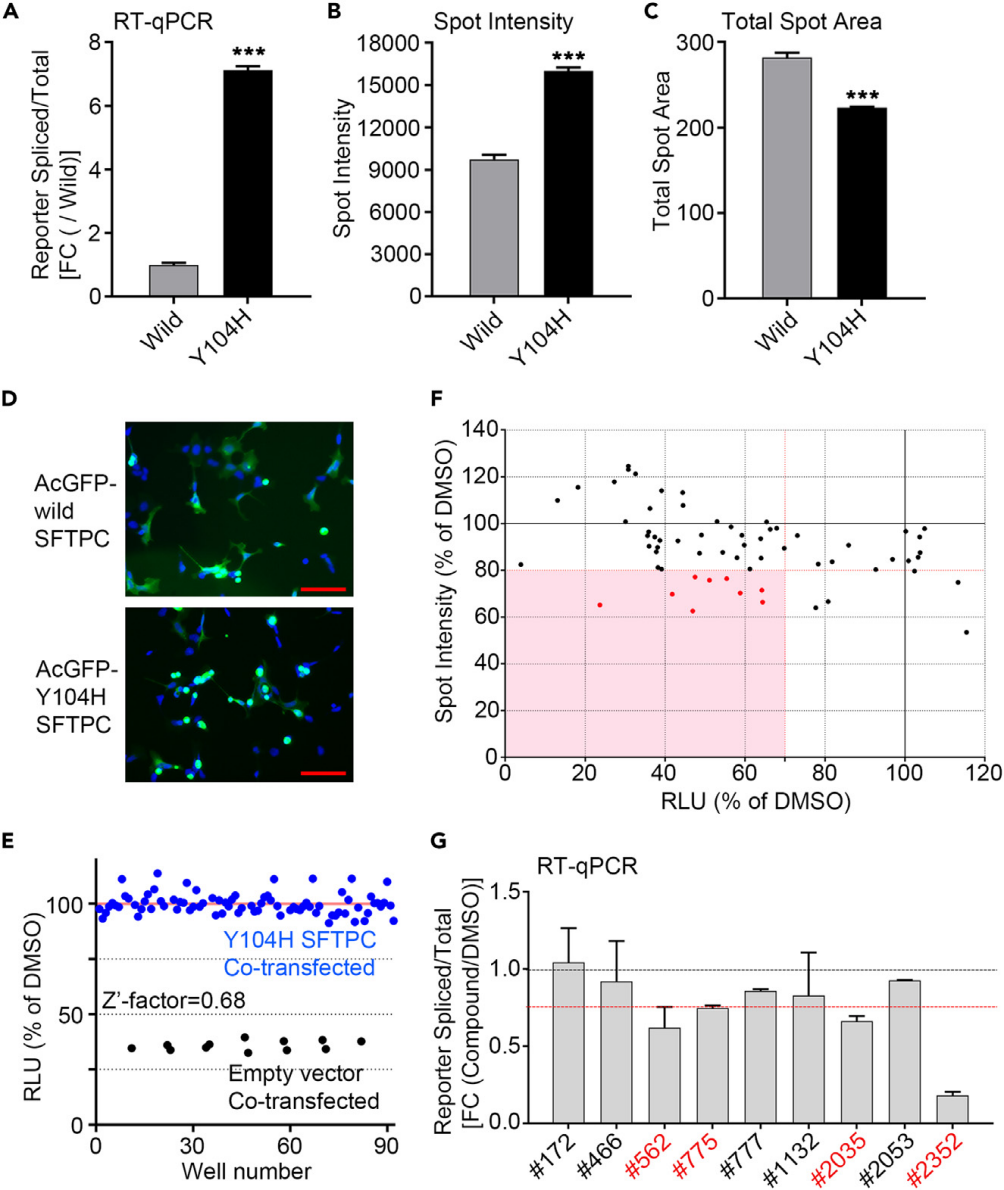
Secondary screening for compounds identified in the primary screening (A) RT-qPCR of the splicing ratio of XBP1-HiBiT Reporter (spliced/total expression) in HEK293 cells subjected to 24 h incubation with co-transfected XBP1-HiBiT Reporter and wild or Y104H SFTPC expression vectors. Data are presented as mean ± SD (n = 3 independent experiments). ∗∗∗p < 0.001 (Student’s *t* test). (B and C) The means of Spot Intensity (B) and Total Spot Area (C) for HEK293 cells transfected with the AcGFP-wild SFTPC or AcGFP-Y104H SFTPC (n = 4, each sample is averaged over 81 field-of-view × 3 well). Data are presented as mean ± SD. ∗∗∗p < 0.001 (Student’s *t* test). (D) Representative microscopic images of HEK293 cells transfected with the AcGFP-wild SFTPC or AcGFP-Y104H SFTPC. Nuclei were visualized by Hoechst 33342 (blue). Scale bar = 100 μm. (E) Validation of screening using cellular HiBiT assay. HEK293 cells were co-transfected with XBP1-HiBiT Reporter and empty or Y104H-mutated *SFTPC* expression vector (Y104H SFTPC). n = 80 (Y104H SFTPC), n = 12 (empty vector). (F) Scatterplot showing pairwise comparisons of the result of the means of Spot Intensity (% of DMSO) for HEK293 cells transfected with the AcGFP-Y104H SFTPC (Data are presented as the mean of n = 2, each sample is averaged over 81 field-of-view) versus the means of RLU (% of DMSO) analyzed by XBP1-HiBiT Reporter co-transfected with Y104H SFTPC in the screening of 65 compounds identified in primary screening (n = 2). Red points indicate the compounds which have passed this screening. (G) Analysis of the splicing ratio of XBP1-HiBiT Reporter (spliced/total expression) by RT-qPCR in HEK293 cells subjected to 4 h incubation with co-transfected XBP1-HiBiT Reporter and Y104H SFTPC expression vectors followed by treatment with each of 9 compounds for 24 h at the indicated concentration (n = 2). Data are presented as mean ± SD. Red points indicate the compounds which have passed this screening. The Red line is FC = 0.75. See also Figure S1.

As a second screening step, we tested whether the candidate compounds could reduce ER stress caused by *SFTPC* mutations other than L188Q. In addition, we simultaneously evaluated whether they could decrease the aggregate formation of mutant *SFTPC* to confirm their effect from a different molecular aspect. As a mutation other than L188Q, we chose the Y104H mutation (c.310T>C), a substitution of tyrosine for histidine at amino acid 104 in the BRICHOS domain of *SFTPC*, because a patient with Y104H was reported in Japan^25^ and patient-derived peripheral blood mononuclear cells (PBMCs) were available for the present study. Therefore, SFTPC expression vectors with the Y104H mutation (Y104H SFTPC) were constructed. First, we investigated whether Y104H SFTPC mutation induces ER stress in the cells similar to L188Q SFTPC mutation. The splicing ratio of the XBP1-HiBiT Reporter was significantly higher in HEK293 cells co-transfected with Y104H SFTPC and the reporter compared with those co-transfected with wild SFTPC and the reporter (Figure 2A). The changes in protein and mRNA expression levels of ER stress marker genes were analyzed in HEK293 overexpressing Y104H SFTPC. In RT-qPCR analysis, mRNAs levels of *BIP*, *CHOP,* and *ATF4,* and endogenous *XBP1* splicing ratio were significantly increased in Y104H SFTPC overexpressed cells relative to wild SFTPC-overexpressed cells (Figure S1C). In WB analysis, BIP and CHOP protein levels were significantly increased in Y104H SFTPC overexpressed cells relative to wild SFTPC-overexpressed cells (Figure S1D). Transfection efficiency was assumed to be almost the same in light of the similar expression level of the neomycin-resistance gene in the two conditions (Figure S1D). These results show that the overexpression of Y104H SFTPC induces ER stress. Next, to analyze whether the Y104H-mutant *SFTPC* causes SFTPC aggregates, each expression vector of the fusion genes of AcGFP and wild or Y104H SFTPC (AcGFP-wild SFTPC and AcGFP-Y104H SFTPC) was generated and transfected into HEK293 cells. Using Opera Phenix high-content screening system, we quantified the mean of the Spot Intensity of AcGFP in each cell (Spot Intensity) and the mean of the Total Spot Area of AcGFP in each cell (Total Spot Area) as an indicator of SFTPC aggregates. We found that the cells transfected with AcGFP-Y104H SFTPC showed significantly higher Spot Intensity than that of AcGFP-wild SFTPC (FC: Y104H/wild = 1.64) and significantly smaller Total Spot Area than that of AcGFP-wild SFTPC (FC: Y104H/wild = 0.79) (Figures 2B–2D). Stronger and smaller GFP spots suggested that the Y104H SFTPC forms aggregates. Because the Z′-factors of the assays, based on the RLT of XBP1-HiBiT Reporter and Spot Intensity of GFP, were high enough for screening [Z′-factor = 0.68 (reporter) and 0.72 (Spot Intensity)] (Figures 2B and 2E), the screening of 65 compounds was performed to determine whether they could reduce ER stress and aggregate formation caused by Y104H-mutant SFTPC. We obtained nine hit compounds with thresholds of 70% RLU and 80% Spot Intensity (% of DMSO) (Figure 2F). To exclude the possibility that these hit compounds affect the CMV promoter or reporter expression system, we analyzed the splicing ratio of the XBP1-HiBiT Reporter for RNA expression levels. The reporter splicing products were detected by RT-qPCR instead of the HiBiT assay and normalized to total reporter expression. Then, four compounds were selected with a threshold of 0.75 for fold change (FC) (compound/DMSO) (Figure 2G).

### Efficacy evaluation of compounds using alveolar epithelial cells differentiated from patient-derived iPSC

**Figure 3:**
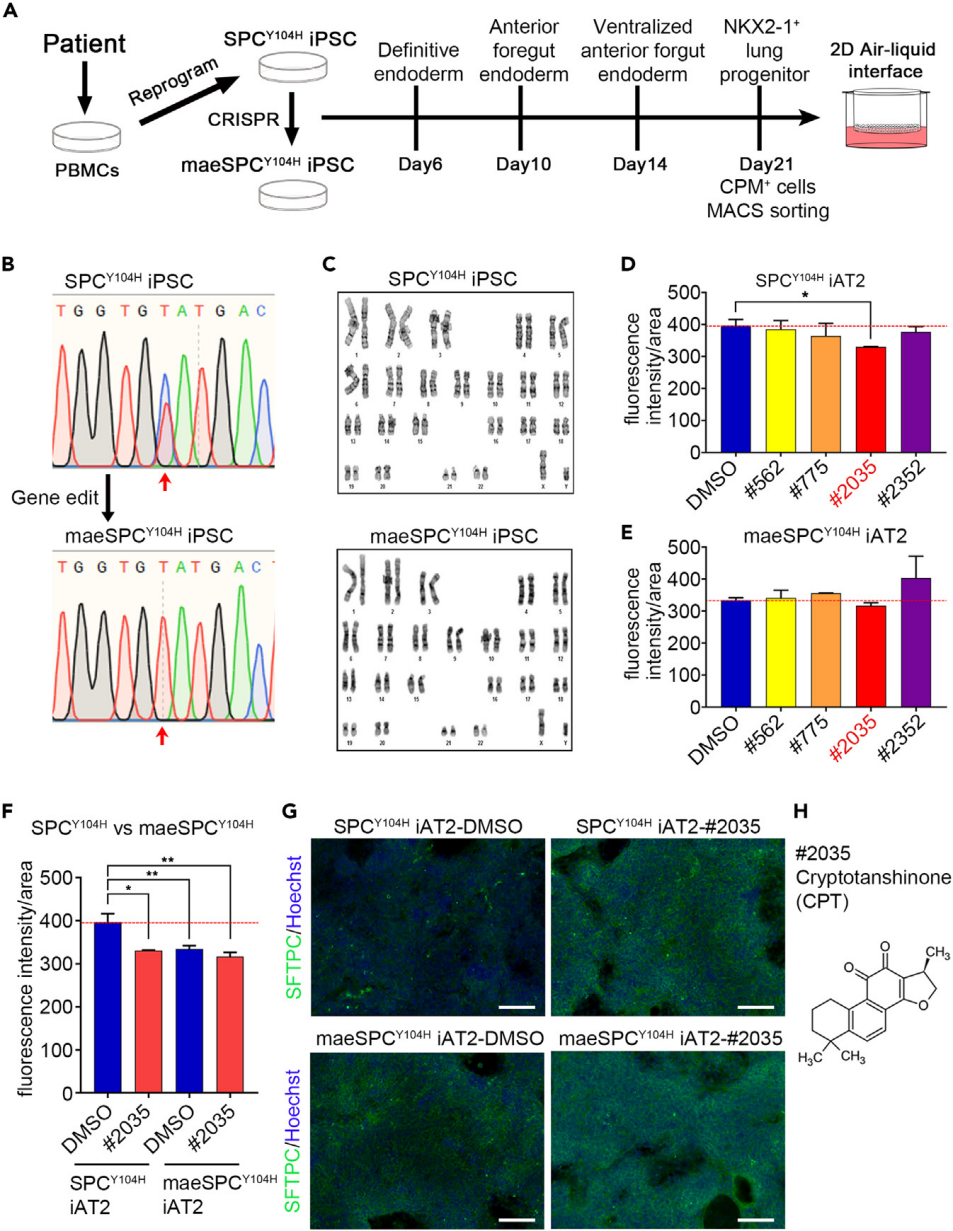
Efficacy evaluation of compounds using alveolar epithelial cells differentiated from the patient-derived iPSC (A) Schematic overview of generation of the patient-derived iPSC (SPC^Y104H^-iPSC) and their isogenic control of wild-type *SFTPC* monoallelic expressing iPSC (maeSPC^Y104H^-iPSC) and their stepwise differentiation into alveolar epithelial cells containing AT2 cells. (B) The sequence analysis of the RT-PCR product from *SFTPC* mRNA at 6 days after induced differentiation into AT2 cells from SPC^Y104H^- and maeSPC^Y104H^-iPSC. Red arrows indicate the position of the Y104H-mutation (c.310T>C). (C) G-banding analysis for the SPC^Y104H^ iPSC and maeSPC^Y104H^-iPSC karyotypes. (D and E) Immunological fluorescence analysis for quantifying SFTPC accumulation in SPC^Y104H^-iAT2 (D) and maeSPC^Y104H^-iAT2 (E) cells differentiated from SPC^Y104H^ iPSC and maeSPC^Y104H^ iPSC. The iAT2 cells in each condition were treated with indicated compounds for 48 h post-7-day differentiation. The fluorescence signal intensity of the high-intensity area of SFTPC staining was quantified and normalized by the fluorescence area. Data are presented as mean ± SD (n = 3 independent experiments). ∗p < 0.05 (Student’s or Welch’s *t* test, compared with DMSO). (F) Comparative analysis of SPC^Y104H^-iAT2 and maeSPC^Y104H^-iAT2 cells treated with CPT based on Figures 3D and 3E results. ∗p < 0.05, ∗∗p < 0.01 (Student’s or Welch’s *t* test, compared with SPC^Y104H^-iAT2 treated by DMSO). (G) Representative image of immunostaining for SFTPC (green) and nuclear counterstaining with Hoechst 33342 (blue) in SPC^Y104H^-iAT2 and maeSPC^Y104H^-iAT2 cells treated with CPT for 48 h post-7-day differentiation. Scale bar = 100 μm. (H) Structure of Cryptotanshinone (CPT), compound number #2035. See also Figure S2 and Table S1.

To examine the efficacy of the compounds identified by the developed screening system, we generated iPSC lines from PBMCs of a patient with ILD who had a heterozygous Y104H-*SFTPC* variant (SPC^Y104H^-iPSC) (Figure 3A). The expression of pluripotency markers (OCT3/4 and NANOG) was confirmed by flow cytometry to assess the pluripotency of SPC^Y104H^-iPSC. The differentiation potential into the three germ layers was determined by ensuring the expression of each differentiation marker [ectoderm (PAX6 and NESTIN), mesoderm (NCAM and Brachyury), and endoderm (SOX17 and FOXA2)] (Figure S2A). SPC^Y104H^-iPSC formed typically tightly packed round-shaped colonies (Figure S2B). Next, iPSC monoallelically expressing one wild type allele of *SFTPC* was generated from SPC^Y104H^-iPSC using CRISPR/Cas9 (hereinafter, wild type *SFTPC* monoallelic expressing iPSC line is called “maeSPC^Y104H^-iPSC”). This is the resultant founder line that expressed no detectable mRNA of Y104H-mutated *SFTPC* (Figure 3B), owing to an intronic insertion of a neomycin cassette flanked by *LoxP* sites. SPC^Y104H^-iPSC and maeSPC^Y104H^-iPSC demonstrated a normal karyotype, as shown by G-banding analysis (Figure 3C). In addition, there were no indels at the 25 predicted off-target sites (Table S1).

To assess the therapeutic potential of the four candidate compounds (#562, #775, #2035, and #2352), SPC^Y104H^-, and maeSPC^Y104H^-iPSC were differentiated into AT2 cells in an air-liquid interface (ALI) culture (hereinafter, these differentiated alveolar epithelial cells containing AT2 cells are called “SPC^Y104H^-iAT2” and “maeSPC^Y104H^-iAT2”) (Figure 3A). The expression of pro-SFTPC in SPC^Y104H^- and maeSPC^Y104H^-iAT2 was confirmed by immunostaining with an anti-proSFTPC antibody (Figure S2C), and quantitative image analysis of SFTPC accumulation was performed. As an indicator of SFTPC accumulation, fluorescence intensity/area scores were calculated by dividing the integrated values of fluorescence intensity by the fluorescence area’s total value. The score of SPC^Y104H^-iAT2 treated with #2035 was significantly lower than that of cells treated with DMSO, although the other compounds showed no significant differences compared with DMSO (Figure 3D). In contrast, the score of maeSPC^Y104H^-iAT2 treated with #2035 showed no significant change (Figure 3E). The fluorescence intensity/area of SPC^Y104H^-iAT2 was higher than that of maeSPC^Y104H^-iAT2, and interestingly, the reduced fluorescence intensity/area of SPC^Y104H^-iAT2 treated by the #2035 was almost the same as that of maeSPC^Y104H^-iAT2 treated with DMSO and #2035 (Figures 3F and 3G). Compound #2035 is identical to CPT (Figures 3H and S2D). These results suggest that CPT contributed to improving SFTPC aggregates caused by the Y104H-mutant SFTPC.

### CPT reduces aggregates and cell death caused by Y104H SFTPC

**Figure 4:**
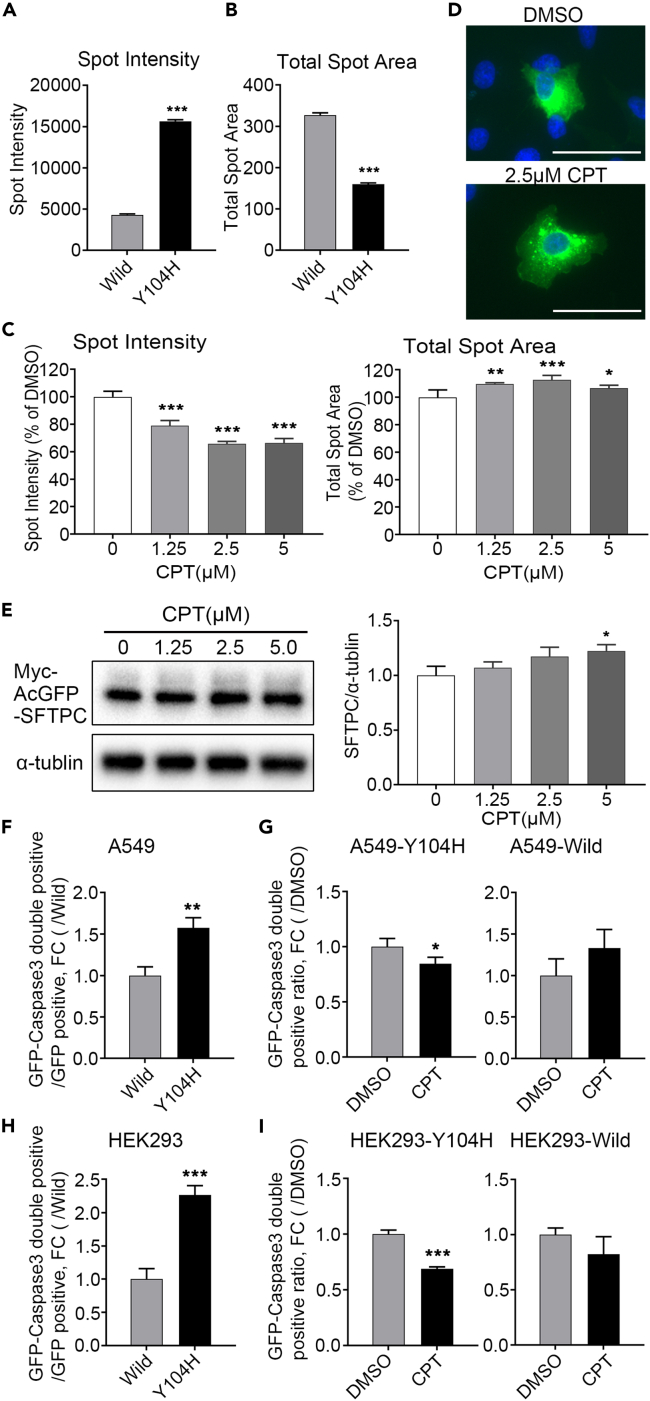
CPT reduces aggregates and cell death caused by Y104H SFTPC (A and B) The means of Spot Intensity of AcGFP in each cell (A) and Total Spot Area of AcGFP in each cell (B) were analyzed for A549 cells transfected with the AcGFP-wild SFTPC or AcGFP-Y104H SFTPC for 48 h (n = 3, each sample is averaged over 81 fields of view). Data are presented as the mean ± SD. ∗∗∗p < 0.001 (Student’s *t* test). (C) Analysis of Spot Intensity and Total Spot Area of A549 cells transfected with the AcGFP-Y104H SFTPC for 4 h and subsequently treated with CPT (1.25, 2.5, or 5 μM) or DMSO (0.1%) for 48 h (n = 3, each sample is averaged over 81 fields of view). Data are presented as the mean ± SD. ∗p < 0.05, ∗∗p < 0.01, ∗∗∗p < 0.001 (One-way ANOVA with Tukey’s multiple comparisons test, compared with DMSO control). (D) Representative image of A549 cells transfected with AcGFP-Y104H SFTPC for 4 h and subsequently treated with CPT (2.5 μM) or DMSO (0.1%) for 48 h. Nuclei were visualized by Hoechst 33342 (blue). Scale bar = 50 μm. (E) Representative results of WB using anti-SFTPC antibody on A549 cells transfected with the AcGFP-wild SFTPC or AcGFP-Y104H SFTPC for 4 h and subsequently treated with CPT (1.25, 2.5, or 5 μM) or DMSO (0.1%) for 48 h. α-tubulin was used as a loading control (Left panel). The signal intensities were quantified using densitometry (Right panel). Three independent experiments were conducted, and the data are presented as the mean ± SD. ∗p < 0.05 (Student’s *t* test, compared with DMSO control). (F) The number of double-positive cells of activated caspase-3 and SFTPC-GFP in A549 cells transfected with the AcGFP-wild SFTPC or AcGFP-Y104H SFTPC for 24 h and calculated by normalizing the number of GFP-positive cells (n = 3, each sample is averaged over 169 fields of view). Data are presented as the mean ± SD. ∗∗p < 0.01 (Student’s *t* test). (G) The number of double-positive cells of activated caspase-3 and SFTPC-GFP in A549 cells transfected with the AcGFP-Y104H SFTPC (Left graph) or AcGFP-wild SFTPC (Right graph) for 4 h and subsequently treated with 1.25 μM CPT or DMSO (0.1%) for 24 h, and calculated by normalizing the total cell number (n = 3, each sample is averaged over 169 fields of view). Data are presented as the mean ± SD. ∗p < 0.05 (Student’s *t* test). (H) The number of double-positive cells of activated caspase-3 and SFTPC-GFP in HEK293 cells transfected with the AcGFP-wild SFTPC or AcGFP-Y104H SFTPC for 24 h and calculated by normalizing the number of GFP-positive cells (n = 3, each sample is averaged over 169 fields of view). Data are presented as the mean ± SD. ∗∗∗p < 0.001 (Student’s *t* test). (I) The number of double-positive cells of activated caspase-3 and SFTPC-GFP in HEK293 cells transfected with the AcGFP-Y104H SFTPC (Left graph) or AcGFP-wild SFTPC (Right graph) for 4 h and subsequently treated with 2.5 μM CPT or DMSO (0.1%) for 24 h, and calculated by normalizing the total cell number (n = 3, each sample is averaged over 169 fields of view). Data are presented as the mean ± SD. ∗∗∗p < 0.001 (Student’s *t* test). See also Figure S3.

To evaluate the effect of CPT on mutant SFTPC in more detail, we analyzed the aggregates of mutant SFTPC for two parameters (intensity and area of spot) and concentration dependence for CPT using A549 cells, which are generally used as lung lineage cells. Spot intensity was significantly higher in A549 cells transfected with AcGFP-Y104H SFTPC than in those transfected with AcGFP-wild SFTPC (FC: Y104H/Wild = 3.6) (Figure 4A). Total Spot Area of AcGFP was significantly smaller in A549 cells transfected with AcGFP-Y104H SFTPC than in those transfected with AcGFP-wild SFTPC (FC: Y104H/Wild = 0.5) (Figure 4B). These results indicate that the GFP spot formed by the transfection of AcGFP-Y104H SFTPC was stronger and smaller than that of AcGFP-wild SFTPC. In A549 cells transfected with AcGFP-Y104H SFTPC and treated with CPT, Spot Intensity decreased, and the Total Spot Area mildly increased in a concentration-dependent manner (IC50 for Spot Intensity: 1.21 μM) (Figures 4C and 4D). These results were similar in HEK293 cells (Figure S3A) and suggested that CPT could diffuse the aggregates of mutant SFTPC in A549 cells. Given the possibility that the decrease in protein levels causes the decrease in intensity, we performed WB to quantify the level of SFTPC protein. The amount of SFTPC proteins was not reduced by CPT treatment (Figure 4E). The mutation of the SFTPC BRICHOS domain can lead to the formation of abnormal intermolecular disulfide bonds, forming aberrant oligomers, which are associated with aggregation.^21^ Therefore, we investigated the aberrant oligomerization caused by Y104H SFTPC and whether CPT affects it. In WB analysis using electrophoresis under non-reducing conditions for AcGFP-Y104H SFTPC, many upward-shifted bands were detected compared with that of AcGFP-wild SFTPC. In particular, large disulfide-linked oligomeric species remained at the top of the gel (Figure S3B). Transfection efficiency was assumed to be almost the same in light of the similar expression level of the neomycin-resistance gene between wild and Y104H (Figure S3C). Since the formation of abnormal oligomerization by Y104H SFTPC was confirmed, we investigated the effect of CPT on this. Concurrently, we examined the impact of CPT on quality control systems such as the ubiquitin-proteasome system and autophagy using MG132 and Bafilomycin A1. No change in the band pattern in the WB analysis of AcGFP-Y104H SFTPC was observed by CPT treatment, either by CPT treatment under MG132 or Bafilomycin A1 (Figure S3D), indicating that CPT does not affect quality control mechanisms such as protein degradation or the formation of abnormal disulfide bonds during folding. These results support that CPT affects the formation or the dissolution of aggregates. Since aggregates of mutant SFTPC are known to result in cell death,^14^^,^^26^ we hypothesized that cell death is reduced by suppressing SFTPC-aggregates using CPT treatment. To verify this hypothesis, we performed an assay for detecting activated caspase-3, an effector caspase that induces apoptosis. The ratio of activated caspase-3 positive cells was higher in A549 cells overexpressing AcGFP-Y104H SFTPC compared with A549 cells overexpressing AcGFP-wild SFTPC, indicating that the expression of Y104H SFTPC induces apoptosis in A549 cells [FC (Y104H/Wild) = 1.57] (Figure 4F). Furthermore, the number of apoptotic cells expressing AcGFP-Y104H SFTPC was significantly reduced by CPT treatment [FC (CPT/DMSO) of Y104H = 0.85], and there was no significant difference in cells expressing AcGFP-wild SFTPC (Figure 4G). These results were similar in HEK293 cells [FC (Y104H/Wild) = 2.27, FC (CPT/DMSO) of Y104H = 0.69] (Figures 4H and 4I). This indicates that CPT suppressed cell death caused by overexpression of the Y104H-mutant SFTPC.

Dose-dependent cytotoxic effect of CPT was confirmed by WST-8 assay. Compared with DMSO, 1.25 μM CPT showed no obvious growth inhibitory effect within 48 h. However, the growth and viability of A549 cells treated with CPT for 24–48 h were significantly inhibited at concentrations >2.5 μM (Figure S3E), consistent with previous reports.^27^ For HEK293 cells, CPT showed no noticeable growth inhibitory effect within 48h and at concentrations <5.0 μM (Figure S3F).

### CPT ameliorates the disease phenotype of alveolar organoids derived from SPC^Y104H^-iPSC

**Figure 5:**
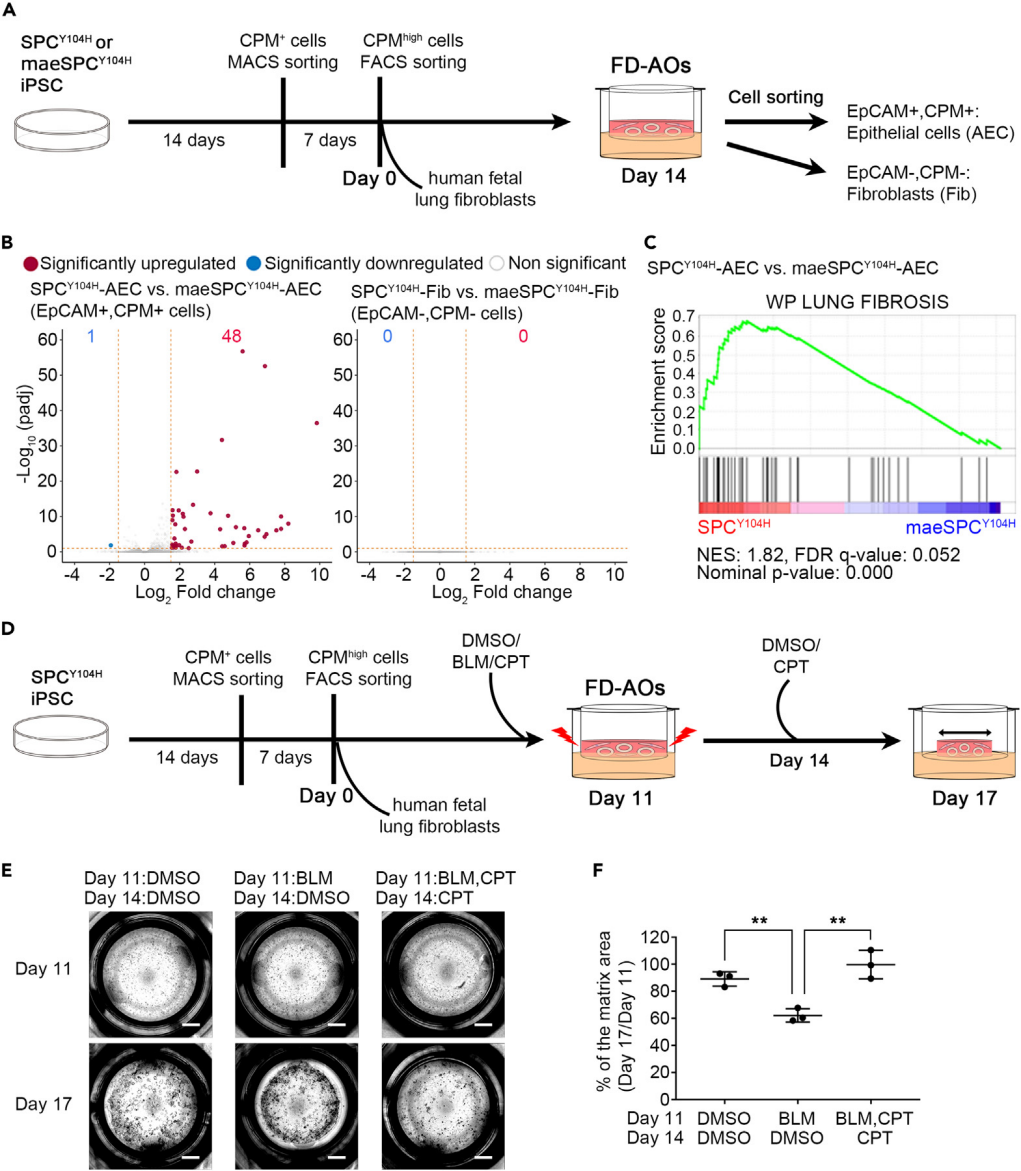
Characterization of FD-AOs generated from SPC^Y104H^ iPSC and its application to BLM-induced contraction assay for validating the efficacy of CPT (A) Schematic overview of generation of the fibroblast-dependent alveolar organoids (FD-AOs) and RNA-seq analysis. (B) Volcano plot generated from the results of DESeq2 analysis. Left panel: SPC^Y104H^-AEC vs. maeSPC^Y104H^-AEC, Right panel: SPC^Y104H^-Fib vs. maeSPC^Y104H^-Fib (n = 3 independent experiments, respectively). AEC and Fib were separated from FD-AOs derived from iPSC, respectively. Red character indicates the number of significantly upregulated genes and blue character indicates the number of significantly downregulated genes. (C) GSEA enrichment plot of the ranked gene expression data by “WP LUNG FIBROSIS” gene set in SPC^Y104H^- and maeSPC^Y104H^-AEC. NES, normalized enrichment score; NOM, nominal; FDR, false discovery rate. (D) Schematic overview of the generation of FD-AOs and their application to the bleomycin (BLM)-induced pulmonary fibrosis model to analyze the efficacy of CPT. (E) Representative whole-well imaging of the cultivation matrices at day 17. Each well was treated with BLM, BLM, and 10 μM CPT or DMSO from day 11 to day 14 and with 10 μM CPT or DMSO from day 14 to day 17. Scale bars, 2 mm. (F) Quantifying matrix area changes on Day 17 (% of Day 14, n = 3 independent experiments, each sample is averaged over three wells). Data are presented as the mean ± SD. ∗∗p < 0.01 (One-way ANOVA with Tukey’s multiple comparisons test). See also Figure S4 and Table S2.

To investigate the efficacy of CPT for the phenotypes of alveolar organoids generated from the patient-derived iPSC, we conceived to combine the *in vitro* pulmonary fibrosis model using the fibroblast-dependent alveolar organoids (FD-AOs) consisting of human pluripotent stem cell-derived alveolar epithelial cells (AEC) and human fetal lung fibroblasts (Fib)^28^ and the patient-derived iPSC. First, to characterize FD-AOs, we performed RNA sequencing (RNA-seq) of AEC and Fib isolated from FD-AOs generated from SPC^Y104H^- and maeSPC^Y104H^-iPSC (hereinafter, these AEC and Fib isolated from FD-AOs generated from SPC^Y104H^-iPSC are called “SPC^Y104H^-AEC” and “SPC^Y104H^-Fib”, and those generated from maeSPC^Y104H^-iPSC are called “maeSPC^Y104H^-AEC” and “maeSPC^Y104H^-Fib”) (Figure 5A). We found 48 upregulated and 1 downregulated differentially expressed genes (DEGs) when comparing SPC^Y104H^-AEC with maeSPC^Y104H^-AEC (padj <0.1 and log2 FC > 1.5 or < −1.5), with no apparent differences in their co-cultured Fib (Figure 5B). In addition, there were 178 upregulated genes in SPC^Y104H^-AEC (padj <0.1 and log2 FC > 0.5), including several genes reportedly associated with lung fibrosis (Table S2). Next, to investigate the downstream consequences of SFTPC Y104H mutation, we performed Gene Set Enrichment Analysis (GSEA) on RNA-seq transcriptome data from SPC^Y104H^- and maeSPC^Y104H^-AEC, respectively, and found that various pathways of inflammatory responses were upregulated in SPC^Y104H^-AEC (Figure S4A). Furthermore, lung fibrosis- and some protein folding-related gene sets were upregulated in SPC^Y104H^-AEC (Figures 5C and S4B). These results indicate that the expression of Y104H SFTPC caused early transcriptomic changes, including the impairment of AEC and the pathway related to protein folding. Given the no significant changes in gene expression in Fib, it is suggested that these alterations in AEC were not sufficient to activate Fib. To analyze the effect of CPT on the impairment of AEC, we selected *integrin subunit beta 6* (*ITGB6*), which is an epithelial-specific receptor, upregulated in response to epithelial injury and highly expressed in idiopathic pulmonary fibrosis lungs, particularly in AT2 cells in the abnormal epithelial cells called “aberrant basaloid cells”.^28^^,^^29^ *ITGB6* was significantly upregulated in SPC^Y104H^-AEC than maeSPC^Y104H^-AEC (Figure S4C). CPT reduced the mRNA level of *ITGB6* in FD-AOs generated from SPC^Y104H^-iPSC in each of the three independent experiments (Figure S4D), suggesting that CPT may ameliorate the impairment of AEC associated with SFTPC mutation.

Furthermore, to examine the efficacy of CPT on alveolar organoid phenotypes, we enhanced the injury of AEC by supplementing bleomycin (BLM) and performed a gel contraction assay, which allows the evaluation of the contraction of the cultivation matrix dependent on the alveolar epithelial-mesenchymal interaction.^28^ SPC^Y104H^-iPSC-derived FD-AOs were prepared and subjected to each treatment with DMSO, BLM, or BLM and CPT (Figure 5D). BLM caused significantly stronger contraction of the gels than DMSO, and CPT suppressed the BLM-induced contraction (Figures 5E and 5F). Then, SPC^Y104H^-AEC were isolated from FD-AOs treated by DMSO, BLM, or BLM and CPT to ask whether the ameliorative effect of CPT on BLM-induced gel contraction is attributable to AEC. RT-qPCR revealed that the expression of *ITGB6* in SPC^Y104H^-AEC was increased by BLM treatment and decreased by CPT treatment in each of the three independent experiments (Figure S4E), suggesting that CPT can suppress BLM-enhanced injury of AEC.

## Discussion

This study has shown that CPT is a potential therapeutic agent in the early stages of SFTPC-associated ILD. CPT was identified throughout the screening system based on two cellular phenotypes caused by mutant *SFTPC*, ER stress, and aggregate formation. The suppressive effect of CPT on mutant SFTPC aggregate formation was evaluated using AT2 cells differentiated from patient-derived iPSC and analyzed in detail using A549 cells. In addition, CPT ameliorated the global contraction of the matrix in FD-AOs with *SFTPC* mutation and decreased cell death of A549 cells and HEK293 cells overexpressing mutant SFTPC.

Growing evidence supports a link between ER stress and lung fibrosis.^30^ ER stress caused by *SFTPC* BRICHOS mutations has been linked to cytotoxicity and is associated with ILD pathogenesis.^9^^,^^13^ In transgenic mice inducibly expressing L188Q-mutant *SFTPC* in AT2 cells, induction of ER stress and exaggerated lung fibrosis after bleomycin treatment was observed.^13^ Therefore, we created a luminescent reporter to quantify ER stress and developed a high-throughput screening system using co-transfection with the reporter and SFTPC expression vector with an ILD-associated mutation, not a drug such as tunicamycin, to more closely mimic the pathological model of familial ILD. We created an ER stress reporter by utilizing cytoplasmic splicing of *XBP1* but not the promoters of ER stress-inducible genes. This allowed us to normalize the amount of spliced mRNA to the total mRNA expressed from the reporter and exclude compounds related to the expression system, such as direct promoter repression.

To narrow down the candidate chemical compounds for the specific pathogenic target, we focused on mutant SFTPC aggregates in addition to ER stress. Compounds that decrease ER stress and aggregates caused by mutant SFTPC are expected to correct the folding of unfolded mutant SFTPC or suppress the formation of aggregates, such as chemical chaperones.^31^ Therefore, a screening system was developed to rigorously quantify the amount of mutant SFTPC aggregates using high-content screening systems. Following these two screening systems, the compounds were evaluated using AT2 cells differentiated from patient-specific iPSC in an ALI culture. SFTPC accumulation in AT2 cells differentiated from iPSC was assessed by immunostaining. Immunostaining for SFTPC can only be used to evaluate AT2 cells because they specifically express SFTPC. These systems allowed us to identify compounds that can suppress the cytotoxicity of epithelial cells involved in the early stages of ILD.

We further evaluated the effect of CPT on the organoid phenotype. For fibrosis to develop, adult-onset mutations, such as the Y104H mutation, require a second hit, such as viral infection or bleomycin. In the mouse model, expression of L188Q SFTPC did not induce lung fibrosis without BLM stimulus. In the absence of a second hit, such mutations are likely to place AEC in a vulnerable state.^13^^,^^32^^,^^33^ In this study, *ITGB6* and lung fibrosis-related gene sets are upregulated in SPC^Y104H^-AEC compared with maeSPC^Y104H^-AEC, suggesting that SPC^Y104H^-AEC are in a vulnerable state. As a second stimulus to mimic mouse models and patients, we administered BLM to FD-AOs generated from SPC^Y104H^-iPSC. Therefore, we confirmed the effect of CPT in the fibrosis model, which recapitulated the lung tissue of pulmonary fibrosis as the contraction of the cultivation matrix. Their feeder- Fib were derived from a distinct donor’s fetal lung Fib; however, the analysis using the identical donor-derived mesenchymal cells, which we previously established,^34^ might provide the model for the evaluation of drug efficacy more similar to *in vivo*.

As an example of the patient-derived iPSC-derived AEC harboring *SFTPC* mutations that cause pulmonary fibrosis, the I73T mutation has recently been reported, in which aberrant SFTPC trafficking, impaired autophagy, and metabolic reprogramming were observed.^35^ I73T-mutant-SFTPC is a non-BRICHOS mutation with no BRICHOS-mutant-SFTPC features, such as ER stress. The present study is the first report of drug evaluation using AEC differentiated from patient-derived iPSC harboring mutations in the BRICHOS domain of *SFTPC*.

Multiple inflammation-related gene sets were upregulated in SPC^Y104H^-AEC in GSEA, consistently with the enrichment analysis of AEC cells with I73T mutated SFTPC.^35^ Despite the different mechanisms implicated in cell toxicity between BRICHOS and non-BRICHOS mutations of SFTPC, similar gene expression changes in the same pathways were observed in the patient-derived AEC, suggesting that such common early epithelial transcriptomic changes may predict the future development of interstitial pneumonia and comparative analysis of alveolar organoids generated from multiple patient-derived iPSC with various SFTPC mutations would lead to elucidate the pathogenesis of ILD. Although we identified multiple genes with significant expression changes from the separated AEC and Fib in the present study, analyses at single-cell resolution may lead to a better understanding of ILD in the future.

CPT is a lipophilic compound extracted from the root of *Salvia miltiorrhiza* (Danshen). It has multiple pharmacological activities, including anti-tumor, anti-inflammatory, neuroprotective, and anti-metabolic effects.^36^ When considering CPT’s mechanism of action in this study, we found that it ameliorated the formation or the dissolution of aggregates, because CPT reduced aggregation without affecting quality control mechanisms such as protein degradation and disulfide bond formation during folding. Mutations in *SFTPA1*/*A2* and *ABCA3*, associated with ILD, have also been reported to cause protein misfolding.^14^^,^^37^^,^^38^ Accumulation of misfolded proteins is a common feature in many neurodegenerative diseases, including Parkinson’s disease, Huntington’s disease, and amyotrophic lateral sclerosis.^39^ Future research on the effects of CPT on these diseases is promising. CPT and the screening strategy used in this study can be applied to therapies targeting the initial alveolar epithelial injury involved in ILD and other protein-folding diseases.

### Limitations of the study

Upregulation of protein folding–related genes in SPC^Y104H^-AEC suggested that the mutant SFTPC was abnormally folded; however, in AEC generated from SPC^Y104H^-iPSC, the expression of ER stress marker genes, including *BiP* and *CHOP*, did not increase. It is speculated that the activation of ER stress-related pathways by SFTPC mutant proteins was adapted during long-term culture.^40^ Viral infections such as flu may remove this adaptation necessitating us to develop the next evaluation system using patient-derived iPSC.

## STAR★Methods

### Key resources table

**Table:** 

REAGENT or RESOURCE	SOURCE	IDENTIFIER
**Antibodies**		

Mouse anti-Myc-Tag IgG2a (9B11)	Cell Signaling Technology	Cat#2276S; RRID: AB_331783
Rabbit anti-α-Tubulin IgG (11H10)	Cell Signaling Technology	Cat#2125; RRID: AB_2619646
HRP-conjugated anti-Mouse IgG-F(ab')2	Abcam	Cat#ab5887; RRID: AB_955402
HRP-conjugated anti-Rabbit IgG-F(ab')2	GE Healthcare	Cat#NA9340; RRID: AB_772191
Rabbit anti-Pro-SP-C, n-terminal	Seven Hills Bioreagents	Cat#WRAB-9337; RRID: AB_2335890
Rabbit anti-Neomycin Phosphotransferase II IgG	Sigma-Aldrich	Cat#06-747; RRID: AB_310234
Rabbit anti-BiP IgG (C50B12)	Cell Signaling Technology	Cat#3177; RRID: AB_2119845
Mouse anti-CHOP IgG2a (L63F7)	Cell Signaling Technology	Cat#2895/ L63F7; RRID: AB_2089254
Rabbit anti-Caspase-3, Active IgG	Sigma-Aldrich	Cat#C8487; RRID: AB_476884
Alexa Fluor 488-donkey anti-Rabbit IgG(H+L)	Thermo Fisher Scientific	Cat#A11070; RRID: AB_2534114
Alexa Fluor 488-donkey anti-Goat IgG(H+L)	Thermo Fisher Scientific	Cat#A11055; RRID: AB_2534102
Alexa Fluor 647-donkey anti-Mouse IgG (H+L)	Thermo Fisher Scientific	Cat#A31571; RRID: AB_162542
PE-conjugated anti-Brachyury	R&D Systems	Cat#IC2085P; RRID: AB_2271455
PE-conjugated Mouse anti-FOXA2	BD Biosciences	Cat#561589; RRID: AB_10716057
Alexa Fluor 488-conjugated Mouse anti-NANOG	BD Biosciences	Cat#560791; RRID: AB_1937305
Alexa Fluor 647-conjugated Mouse anti-OCT3/4	BD Biosciences	Cat#560329; RRID: AB_1645318
Alexa Fluor 488 conjugated Mouse anti-PAX6	BD Biosciences	Cat#561664; RRID: AB_10895587
Alexa Fluor 647-conjugated Mouse anti-SOX17	BD Biosciences	Cat#562594; RRID: AB_2737670
Alexa Fluor 647-conjugated Mouse IgG1κ isotype control	BioLegend	Cat#400130; RRID: AB_2800436
BV421-conjugated Mouse IgG1κisotype control	BioLegend	Cat#400157; RRID: AB_10897939
Alexa Fluor 488-conjugated Mouse IgG1κ, isotype control	BioLegend	Cat#400132; RRID: AB_2890263
BV421-conjugated Mouse anti-Nestin	BioLegend	Cat#656808; RRID: AB_2566634
Alexa Fluor 488-conjugated Mouse IgG2aκ, isotype control	BD Biosciences	Cat#565362; RRID: AB_2869664
Alexa Fluor 647-conjugated Mouse anti-CD56 (NCAM)	BioLegend	Cat#362513; RRID: AB_2564086
Alexa Fluor 488-conjugatedGoat IgG isotype control	R&D Systems	Cat#IC108G; RRID: AB_10890944
PE-conjugated Mouse IgG isotype control	Beckman Coulter	Cat#A07796; RRID: AB_2832963
Mouse anti-CPM IgG2b (WK)	FUJIFILM Wako	Cat#014-27501; RRID: AB_2801482
Goat anti-EpCAM/TROP-1 IgG	R&D Systems	Cat#AF960; RRID: AB_355745
Rat anti-CPM IgG2a (43A1)	Gotoh Lab	N/A
Mouse anti-Rat Kappa MicroBeads	Miltenyi Biotec	Cat#130-047-401; RRID: AB_244353

**Chemicals, peptides, and recombinant proteins**		

Function-known chemical library	Medical Research Support Center in Kyoto University	N/A
Cryptotanshinone	Tokyo chemical industry	Cat#C3363
Tunicamycin	FUJIFILM Wako	Cat#11089-65-9
Bleomycin	Nippon Kayaku	Cat#14987170006106
Bafilomycin A1	Cayman Chemical Company	Cat#11038
MG-132	Sigma-Aldrich	Cat#474790
Dimethyl sulfoxide	Sigma-Aldrich	Cat#D2650

**Critical commercial assays**		

Nano Glo HiBiT Lytic Detection System	Promega	Cat#N3030
Perm/Wash Buffer	BD Biosciences	Cat#554723

**Deposited data**		

NGS results from SPCY104H or maeSPCY104H iPSC-derived FD-AOs	This Study	NBDC Human Database: JGAS000617 DDBJ: E-GEAD-623

**Experimental models: Cell lines**		

HEK293 human embryonic kidney cell line	ATCC	CRL-1573
A549 Human adeno carcinoma cell line	JCRB	JCRB0076
SPC^Y104H^-iPSC	Saito Lab	Cat#CiRA00995-F
maeSPC^Y104H^-iPSC	Gotoh Lab	CiRA00995-F-Res10-2
Human fetal lung fibroblasts	DV Biologics	Cat#PP002-F-1349

**Oligonucleotides**		

See Primer list		N/A

**Recombinant DNA**		

pGEM-T Easy vector	Promega	Cat#A1360
pGEM_SFTPC_Wild	This paper	N/A
pGEM_SFTPC_L188Q	This paper	N/A
pGEM_SFTPC_Y104H	This paper	N/A
pcDNA3.1-myc-SFTPC-Wild	This paper	N/A
pcDNA3.1-myc-SFTPC-L188Q	This paper	N/A
pcDNA3.1-myc-SFTPC-Y104H	This paper	N/A
pcDNA3.1-AcGFP-SFTPC-Wild	This paper	N/A
pcDNA3.1-AcGFP-SFTPC-Y104H	This paper	N/A
pcDNA3-myc-XBP1-exon4	This paper	N/A
pCE-hOCT3/4	Okita et al.^41^	N/A
pCE-hSK	Okita et al.^41^	N/A
pCE-hUL	Okita et al.^41^	N/A
pCE-mp53DD	Okita et al.^41^	N/A
pCXB-EBNA1	Okita et al.^41^	N/A

**Software and algorithms**		

Harmony software	Perkin Elmer	https://www.perkinelmer.com/
GraphPad Prism Version 8	GraphPad	www.graphpad.com
Hybrid cell count BZ-H3C software	Keyence	https://www.keyence.com/
Image Lab software	Bio-Rad	https://www.bio-rad.com/
FlowJo software	BD Biosciences	https://www.bdj.co.jp
iDEP.96	Ge et al.^42^	http://bioinformatics.sdstate.edu/idep96/
ggVolcanoR 1.0	Mullan et al.^43^	https://ggvolcanor.erc.monash.edu/

**Other**		

Human lung total RNA	Innivative Cell Technologies	Cat#AT-104-500
PBS	Nacalai Tesque	Cat#14249-24
Glutamax	Thermo Fisher Scientific	Cat#35050-061
FuGENE HD Transfection Reagent	Promega	Cat#E2311
Random primers	Takara Bio	Cat#3801
FastStart Universal SYBR-Green Master	Hoffmann-La Roche	Cat#4913850001
Sample Buffer Solution with Reducing Reagent(6x) for SDS-PAGE	Nacalai Tesque	Cat#09499-14
Can Get Signal immunoreaction enhancer solution	Toyobo	Cat#NKB-101
Chemi-Lumi One Super	Nacalai Tesque	Cat#02230-30
ImmunoStar LD	FUJIFILM Wako	Cat#290-69904
Bovine serum albumin	Sigma-Aldrich	Cat#A7906
Hoechst 33342	Nacalai Tesque	Cat#04929-82
Cell Count Reagent SF	Nacalai Tesque	Cat#07553-15
StemFit AK02N	Takara Bio	Cat#AK02N
iMatrix-511	Takara Bio	Cat#T303
STEMdiff™ Trilineage Differentiation Kit	STEMCELL technologies	Cat#ST-05230
Y-27632	LCL Laboratories	Cat#LCL-Y-5301-250
Accutase	Innovative Cell Technologies	Cat#AT104
G418 Solution	Thermo Fisher Scientific	Cat#10131035
mTeSR plus	STEMCELL Technologies	Cat#ST-05825/ST-100-0276
Geltrex	Thermo Fisher Scientific	Cat#A1413302
Dexamethasone	Sigma-Aldrich	Cat#D4902-25MG
KGF	Peprotech	Cat#100-19-250UG
8-Br-cAMP	Life Science Institute	Cat#B007-100
IBMX	FUJIFILM Wako	Cat#099-03411
CHIR99021	AXON Medchem	Cat# AXON1386-25
SB431542	FUJIFILM Wako	Cat#198-16543
Matrigel Growth Factor Reduced Basement Membrane Matrix	Corning	Cat#354230

### Resource availability

#### Lead contact

Shimpei Gotoh (gotoh.shimpei.5m@cira.kyoto-u.ac.jp).

#### Materials availability

Materials used in this study are available upon request under a completed materials transfer agreement. SPC^Y104H^ and maeSPC^Y104H^ iPSC were deposited at RIKEN BRC (Tsukuba, Japan) (HPS5200 and HPS5250, respectively).

### Experimental model and study participant details

#### Ethics

After the written informed consents were obtained from the patient, iPSC were established and subjected to the subsequent study under the approval by Ethics Committee of Kyoto University Graduate School and Faculty of Medicine (R91/G259).

#### Cell culture and transfection

HEK 293 cells (ATCC, Manassas, VA, USA) were cultured in Dulbecco’s Modified Eagle’s Medium (DMEM) containing 10% fetal bovine serum (FBS), 1% penicillin-streptomycin (Nacalai Tesque, Kyoto, Japan), and 1% GlutaMAX (Thermo Fisher Scientific, Waltham, MA, USA). A549 cells (JCRB, Japan) were cultured in Minimum Essential Medium containing 10% FBS and 1% penicillin-streptomycin. Plasmids were transfected with the FuGENE HD Transfection Reagent (Promega, Madison, WI, USA).

#### Plasmids

The XBP1-HiBiT Reporter was constructed using the exon 4 region of *XBP1* gene from RNA extracted from A549 cells, amplified by RT-PCR, and cloned into pcDNA3.1-Myc with primers including the sequences of HiBiT (KP011 and KP012) at the HindIII/XbaI sites. The *SFTPC* expression vectors were cloned into the TA cloning vector pGEM-T Easy Vector (Promega) using human lung total RNA (Z6524N, Takara, Shiga, Japan). The protein sequence was UniProt number P11686-1. The mutation for L188Q and Y104H was introduced by a PCR-based method^44^ using pGEM-T Easy vector (Promega) with primers (L188Q: KP023 and KP024; Y104H: KP114 and KP115). *SFTPC* in pGEM-T easy vector was amplified by PCR using primers (KP049 and KP050) with restriction sites and ligated to the 3’-end of the Myc-tag in pcDNA3.1-Myc at the HindIII/XbaI sites. PcDNA3.1-AcGFP-*SFTPC* (wild type and Y104H) was generated by 3-fragment-ligation at the HindIII/XhoI/XbaI sites. AcGFP was isolated from pAcGFP-N1 (Takara) using PCR with the primers (KP051 and KP052).

### Method details

#### Image analysis

HEK293 cells were plated on 96- or 24-well PureCoat amine-coated plates (Falcon; BD Biosciences, Franklin Lakes, NJ, USA). A549 cells were plated on 24-well plates (Falcon). Analyses were performed using Opera Phenix (Perkin Elmer, MA, USA) with Harmony software (for Spot Intensity, Total Spot Area, and caspase-3 positive cells of HEK 239 and A549 cells) or a fluorescence microscope BZX710 (Keyence, Osaka, Japan) with Hybrid Cell Count software (for fluorescence intensity/area of SPC^Y104H^ and maeSPC^Y104H^ -iAT2 cells).

#### RT-qPCR

First-strand cDNAs were synthesized using random primers (Takara), and qPCR was performed using FastStart Universal SYBR-Green Master (Rox) (Hoffmann-La Roche, Inc., Nutley, NJ, USA) according to the manufacturer’s protocol. The spliced-isoform-specific primer was referenced from the previously reported sequence.^45^ The primer numbers used are shown below:

Spliced XBP1-HiBiT Reporter: KP163 and KP038.
Total expression of XBP1-HiBiT Reporter: KP109 and KP038.

#### Sequence analysis from RT-PCR products

Total RNA extraction, cDNA synthesize and RT-PCR were performed as previously described.^46^ RT-PCR products were purified with Wizard SV Gel and PCR Clean-Up System (Promega) and sequenced. RT-PCR and sequencing were performed with primers (KP125 and KP166).

#### Western blotting (WB)

WB was performed according to the previously reported method.^47^ Proteins were extracted from A549 cells using a sample buffer (Nacalai Tesque), and the lysates were denatured at 95°C for 3 min. The lysates were then resolved by SDS-PAGE (SuperSep Ace gel; FUJIFILM Wako, Osaka, Japan) and transferred to a polyvinylidene difluoride membrane (Pall Corporation, Port Washington, NY, USA). Antibody reactions were performed using the Can Get Signal immunoreaction enhancer solution (Toyobo, Osaka, Japan). Immunoreactivity was visualized using Chemi-Lumi One Super (Nacalai Tesque) or ImmunoStar LD (FUJIFILM Wako) and ChemiDoc MP imaging system (Bio-Rad, Hercules, CA, USA). The antibodies used in this study are listed in the Antibody List.

#### Immunocytochemistry

Differentiated AT2 cells on a cell culture insert were removed with a membrane using biopsy punches (BP-50F) (Kai Corporation, Tokyo, Japan), fixed with 4% paraformaldehyde in PBS, and pre-incubated in PBS with 3% bovine serum albumin (BSA) (Sigma-Aldrich, St. Louis, MO, USA) and 0.1% Triton X-100 for 30 min at 20°C to 25°C. Primary antibodies in 3% BSA/PBS were applied overnight at 4°C, followed by 1 h at 20°C to 25°C with the secondary antibodies. The antibodies used in this study are listed in the antibody list. The nuclei were stained with Hoechst 33342 (Nacalai Tesque). The cells were observed using a fluorescence microscope (model BZX710; Keyence). Analysis was performed using three independent samples. A TCS SP8 (Leica Microsystems) was used for confocal imaging.

#### HiBiT assay

For the HiBiT assay, HEK293 cells (4.0 × 10ˆ5 cells/mL) were seeded and transfected overnight with the XBP1-HiBiT Reporter and SFTPC expression vector using the FuGENE HD Transfection Reagent (Promega). Four hours after transfection, the cells were incubated with each compound of the function-known chemical library provided from Medical Research Support Center in Kyoto University (final concentration was 10 μM) or DMSO for 24 h, followed by reseeding into 96- well PureCoat amine-coated plates (Corning, NY, USA) (4.0 × 10ˆ4 cells/mL). To validate the quantifiability of the reporter, tunicamycin (FUJIFILM Wako) was used at 0.6, 1.25, 2.5, or 5.0 μg/mL for 6 h. Luminescence was subsequently measured using the Nano-Glo HiBiT Lytic Detection System according to the manufacturer’s instructions (Promega). Relative luminescence units (RLUs) were measured using ARVO (PerkinElmer, Waltham, MA, USA). For cells treated in a similar manner, the WST-8 assay was performed by using Cell Count Reagent SF (Nacalai Tesque).

#### Generation and maintenance of patient-derived iPS cells

iPS cells were established from PBMCs as described previously.^41^^,^^48^^,^^49^^,^^50^ Briefly, after the consents were obtained from the patient, PBMCs were isolated using BD Vacutainer Cell Preparation tubes (BD Biosciences) and cryopreserved. Thawed PBMCs were reprogrammed into iPSC clones with episomal plasmids (pCE-hOCT3/4, pCE-hSK, pCE-hUL, pCE-mp53DD, and pCXB-EBNA1), under feeder-free culture in StemFit AK02N medium on iMatrix-511-coated plates (Nippi, Tokyo, Japan) as described previously.^49^ The established iPSC clones were cryopreserved in Stem Cell Banker (Takara Bio) and maintained in StemFit AK02N medium supplemented with 50 U/mL penicillin-streptomycin (Thermo Fisher Scientific). The established SPC^Y104H^-iPSC clone (Clone #CiRA00995-F) was evaluated using a STEMdiff Trilineage Differentiation Kit (STEMCELL Technologies, Vancouver, Canada) as described previously.^48^ In brief, the dissociated cells were reseeded onto culture plates at 2.0 × 10ˆ5 cells for endoderm differentiation and 4.0 × 10ˆ5 cells for ectoderm and mesoderm differentiation, respectively and were differentiated, according to the manufacturer’s instructions. Each sample of three germ layers (1.0x10ˆ6 cells per sample) was fixed with 4% paraformaldehyde in PBS for 20 min at 4°C and washed twice with 2%FBS/PBS. Samples were permeabilized with BD Perm/Wash buffer (BD Biosciences) for 15 min at room temperature and stained with the fluorescence-conjugated antibodies listed in the Antibody List. Then, they were washed with BD Perm/Wash buffer and subjected to flowcytometric analysis using LSR (BD Biosciences). The data was analyzed with FlowJo software (FlowJo, LLC, Ashland, OR, USA). G-banding analysis was performed at Nihon Gene Research Laboratories (Sendai, Miyagi, Japan).

#### Gene edition of SPC^Y104H^ iPSC

After SPC^Y104H^ iPSC were maintained on iMatrix-511-coated plates in StemFit AK02N medium (Ajinomoto, Tokyo, Japan), Y-27632 was supplemented at 10 μM for at least 1 h before electroporation. The cells were dissociated into single cells using Accutase (Innovative Cell Technologies, Inc., San Diego, CA, USA) at 37°C for 20 min and neutralized with DMEM/F12 (Nacalai Tesque) supplemented with 2% FBS (Sigma-Aldrich). Three plasmid vectors comprising 5 μg Cas9, 5 μg sgRNA, and 5 μg donor template with a neomycin-resistant gene cassette were simultaneously electroporated into 1.0 x 10ˆ6 cells using a NEPA 21 electroporator (poring pulse: pulse voltage, 125 V; pulse width, 5 ms; pulse number, 2) (Nepagene, Chiba, Japan), as previously reported.^16^^,^^51^ The cells were then reseeded on iMatrix-511-coated plates in StemFit AK02N medium supplemented with 10 μM Y-27632 for 1-2 day. Two days after electroporation, G418 (Gibco BRL) selection was initiated at 100 μg/ml. After selection for 7-10 day, the cells were dissociated and replated at limiting dilutions of 200, 500, and 1,500 cells per iMatrix-511-coated 10-cm dish. Subclones were selected and screened using genomic PCR. When homologous recombination was suspected, Sanger sequencing was performed. Finally, the maeSPC^Y104H^ iPSC (Clone #CiRA00995-F-Res10-2) having gene-edited *SFTPC* with an intronic insertion of a neomycin cassette flanked by LoxP sites. A clone with a normal karyotype was selected for subsequent G-band chromosomal analysis. The maeSPC^Y104H^ iPSC were verified to be derived from the parental iPS cells using short tandem repeat analysis. The predicted off-target sites with up to 3-bp mismatches were obtained from the GGGenome database (GGGenome https://gggenome.dbcls.jp/en/) and analyzed by Sanger sequencing (Table S1).

#### Differentiation of patient-derived iPS cells into AT2 cells

Before differentiation, SPC^Y104H^ and maeSPC^Y104H^ iPS cells were maintained in a mTeSR Plus medium (STEMCELL Technologies). Each iPS cell line was differentiated stepwise into NKX2-1^+^ lung progenitor cells, as described previously.^52^ NKX2-1^+^ lung progenitor cells were isolated using a magnet-activated cell sorting system (Miltenyi Biotec, Auburn, CA, USA). Briefly, NKX2-1^+^ lung progenitor cells were magnetically labeled with an anti-carboxypeptidase M (CPM) antibody (in-house) and microbead-conjugated secondary antibody (Miltenyi Biotec, Cat# 130-047-401), and CPM^+^ cells were isolated using magnetic columns.^52^^,^^53^ Isolated CPM^+^ cells were seeded onto Geltrex-coated upper chambers of 24-well cell culture inserts (Falcon, #353104), followed by 7-day differentiation into alveolar epithelial cells in ALI culture, and subsequently treated with the compounds (10 μM) or DMSO (0.1%) for 48 h. Alveolar differentiation medium was supplemented with dexamethasone (Sigma-Aldrich, Cat# D4902), KGF (Cat# 100-19; PeproTech, Rocky Hill, NJ, USA), and 8-Br-cAMP (Cat# B007; Biolog, Hayward, CA, USA), 3-Isobutyl-1-methylxanthine (IBMX), CHIR99021 (Cat# 1386; Axon Medchem, Groningen, Netherlands), SB431542 (FUJIFILM Wako, 1342 Cat# 198-16543), and Y-27632 (Cat# Y-5301; LC Laboratories, Woburn, MA, USA) were used to induce alveolar epithelial cells in fibroblast-free conditions, as described previously.^52^ We obtained human AT2 cells from each iPS cell line.

#### Culture of human fetal lung fibroblasts and maintenance of iPS cells-derived AT2 cells in FD-AOs

Human fetal lung fibroblasts (17.5 weeks of gestation; DV Biologics #PP002-F-1349) were cultured in DMEM (Nacalai Tesque) supplemented with 10% fetal bovine serum (FBS; Sigma-Aldrich #F7524) and 50 U/mL penicillin-streptomycin (P-S; Thermo Fisher Scientific). Each iPSC line was differentiated stepwise into NKX2-1^+^ lung progenitor cells, and CPM^+^ cells were isolated using magnetic columns, as described above, on day 14. Seven days later, CPM^high^ cells were further isolated using fluorescence-activated cell sorting (FACS) with mouse anti-CPM antibody (FUJIFILM Wako) as described previously.^16^ FACS-sorted CPM^high^ cells (1 × 10^4^) were mixed with 5 × 10^5^ precultured lung fibroblasts in 200 μL of 50% growth factor reduced Matrigel (Corning #354230) diluted with DCIK medium^52^ supplemented with 10 μM Y-27632 (LC Laboratories). Approximately 200 μL of the mixed cells was placed on a 12-well cell culture insert (Corning), and 1 mL of DCIK medium containing 10 μM Y-27632 was added to the lower chamber. In the 24-well format, half the fluid volume was used. FD-AOs were cultured for 14 days, and the medium in the lower chambers was replaced with DCIK medium every two days. FACS was performed using BD FACS Melody Cell Sorter (Becton Dickinson, San Jose, CA, USA) in this study.

#### Isolation of epithelial cells and fibroblasts from FD-AOs

Matrigel-embedded cells were carefully dissociated at 37°C for 25 min via gentle pipetting with 0.1% Trypsin–EDTA (Thermo Fisher Scientific). Dissociated cells were stained with anti-EpCAM/TROP (Bio-Techne #AF960) and anti-CPM (Wako #014-27501) antibodies. After being washed twice with the flow cytometry buffer, the cells were stained with the following secondary antibodie (see the Antibody List). Flow cytometry was performed to separate AEC (EpCAM+CPM+) and fibroblasts (EpCAM-CPM-) for the downstream analysis.

#### RNA-seq and data analysis

The RNeasy Micro Kit was used to extract the total RNA following the manufacturer’s protocol. The RNA quality of each sample was assessed using a 2100 Bioanalyzer (Agilent Technologies). Sequencing libraries of the AEC and Fib samples were prepared using the Stranded Total RNA Prep, Ligation with Ribo-Zero Plus kit (Illumina). Sequencing was performed on the Illumina NextSeq2000 with paired-end mode (59bp X 2). After trimming adapter sequences and low quality bases from the raw sequenced reads with cutadapt v4.1,^54^ the trimmed reads were aligned to human genome (GRCh38) using STAR 2.7.10a^55^ with the GENCODE (release 32, GRCh38.p13).^56^ The raw counts were calculated using htseq-count ver. 2.02^57^ with the GENCODE gtf file. Read counts were scaled across samples using the web portal for integrated Differential Expression and Pathway analysis (iDEP.91; http://bioinformatics.sdstate.edu/idep/).^42^ DEGs were subsequently quantified using DESeq2^58^ based on padj <0.1 and log2 (fold change) > 1.5 or < −1.5. Volcano plots were created with ggVolcanoR 1.0.^43^

#### GSEA

Expressed genes [CPM (Counts per Million mapped reads)] ≥ 0.5 for at least one library) were used as input for GSEA^59^ using GSEA 4.3.2 with the following parameters: the number of permutations = 1000, metric = Diff_of_Classes, permutation type = gene_set, max_size = 500, min_size = 15, and C2 curated and Hallmark gene sets from the Molecular Signatures Database (https://www.gsea-msigdb.org/gsea/msigdb/index.jsp).

#### BLM and compound treatment in FD-AOs

CPM^high^ cell-derived FD-AOs were treated with 3 μg/mL BLM (Nippon Kayaku, Tokyo, Japan), DMSO (0.1%) or 3 μg/mL BLM, and 10 μM CPT in the lower chamber medium from day 11 to day 14. Compounds were washed out on day 14 with PBS (Nacalai Tesque), and the FD-AOs were cultured in dexamethasone-free “CIK” medium from day 14-17. 10 μM CPT or DMSO (0.1%) was supplemented in the medium from day 14 to day 17. Images were obtained using a BZ-X710 microscope (Keyence). Images were joined, and the whole matrix area was measured using BZX Analyzer (Keyence).

### Quantification and statistical analysis

Values are presented as mean ± SD. Statistical significance was evaluated with a two-tailed Student’s or Welch’s t-test to analyze differences between two experimental groups (p < 0.05 was considered significant). In Figures 1B, 4C, 4E, 5F, S1, S3A, S3E, and S3F statistical significance was evaluated with one-way ANOVA with Tukey's multiple comparisons tests (statistical significance was set at p < 0.05).

#### Primer list

**Table:** 

**For plasmids construction**	

KP023	gtgtggcgaggtgccgctctac
KP024	Tgggtgctcacggccatgccc
KP049	TTTAAGCTTGGTGGTCTCGAGatggatgtgggcagcaaagagg
KP050	TTTTCTAGActagatgtagtagagcggcacc
KP051	TTTAAGCTTatggtgagcaagggcgccgagc
KP052	TTTCTCGAGtcCcttgtacagctcatccatgc
KP114	tttaagcttatggtgagcaagggcgccgagc
KP115	tttctcgagtcccttgtacagctcatccatgc

**For RT-PCR sequence for *SFTPC* mRNA**	

KP125	tggggcgccggaagcccagc
KP166	gatgctctctggagctatcttc

**For RT-qPCR**	

KP163 (Spliced *XBP1* reporter-F)	gctgagtccgcagcaggt
KP038 (Spliced *XBP1* reporter-R)	cgaaatcttcttgaacagccgcc
KP109 (Total expression reporter-F)	tgcaggcccagttgtcac
KP038 (Total expression reporter-R)	cgaaatcttcttgaacagccgcc
KP257 (BIP-F)	taaacccagatgaagctgtagcg
KP258 (BIP-R)	acacctcccacagtttcaatacc
KP305 (CHOP-F)	ctggtatgaggacctgcaagag
KP306 (CHOP-R)	cagagaagcagggtcaagagtg
KP303 (ATF4-F)	cttcaaacctcatgggttctcc
KP304 (ARF4-R)	ctccaacatccaatctgtcccg
KP255 (ITGB6-F)	atattgacacacccgaaggtgg
KP256 (ITGB6-R)	atcactcacaaagaccaggagg

## Data Availability

Data reported in this paper will be shared by the lead contact upon request. NGS results from SPC^Y104H^ or maeSPC^Y104H^ iPSC-derived FD-AOs are available at DNA Data Bank of Japan (DDBJ). Accession numbers are listed in key resources table. This paper does not report original code. Any additional information required to reanalyze the data reported in this paper is available from the lead contact upon request.

## Supplemental information

Document S1. Figures S1–S4 and Table S1

Table S2. Genes upregulated in SPC^Y104H^-AEC, related to Figure 5
