# Supplementary material for: Cryptotanshinone is a candidate therapeutic agent for interstitial lung disease associated with a BRICHOS-domain mutation of SFTPC
Source: iScience. 2023 Aug 25;26(10):107731. doi: 10.1016/j.isci.2023.107731 (PMC10494175; doi:10.1016/j.isci.2023.107731)
Supplement: Document S1. Figures S1–S4 and Table S1 [file mmc1.pdf]

iScience, Volume *26*

Supplemental information

Cryptotanshinone is a candidate therapeutic agent for interstitial lung disease associated with a BRICHOS-domain mutation of *SFTPC*

Motoyasu Hosokawa, Ryuta Mikawa, Atsuko Hagiwara, Yukiko Okuno, Tomonari Awaya, Yuki Yamamoto, Senye Takahashi, Haruka Yamaki, Mitsujiro Osawa, Yasuhiro Setoguchi, Megumu K. Saito, Shinji Abe, Toyohiro Hirai, Shimpei Gotoh, and Masatoshi Hagiwara

SUPPLEMENTAL FIGURES AND LEGENDS

Figure S1

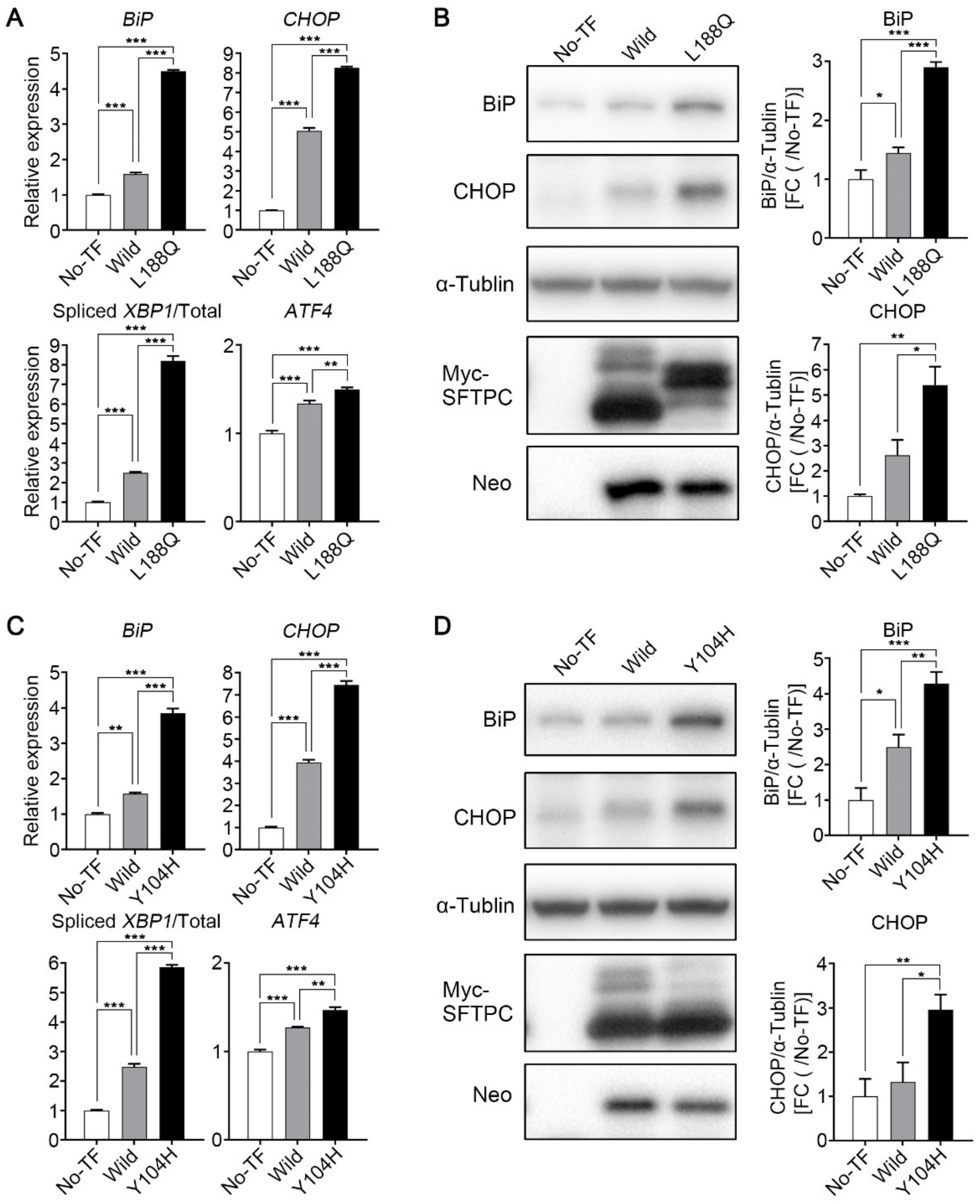

Figure S1. ER stress induction in HEK293 cells expressing L188Q or Y104H SFTPC, related to Figures 1 and 2.

(A) RT-qPCR of indicated gene expression in HEK293 cells co-transfected with wild or L188Q SFTPC and XBP1-HiBiT Reporter or no transfection cells (No-TF) (n = 3). Data are presented as mean ± SD. $^{**}p < 0.01$, $^{***}p < 0.001$ [one-way analysis of variant (ANOVA) with Tukey's

multiple comparisons test]. **(B)** Representative results of WB for indicated proteins in HEK293cells co-transfected with wild or L188Q SFTPC and XBP1-HiBiT Reporter or no transfection cells (No-TF). Signal intensities of BiP and CHOP in WB were quantified by densitometry, normalized by α-Tubulin, and indicated using a bar graph (n = 3). Neo, neomycin phosphotransferase II (neomycin-resistance gene). Data are presented as mean ± SD. *$p < 0.05$, **$p < 0.01$, ***$p < 0.001$ (one-way ANOVA with Tukey's multiple comparisons test). **(C)** RT-qPCR of indicated gene expression in HEK293 cells transfected with wild or Y104H SFTPC, or No-TF (n = 3). Data are presented as mean ± SD. **$p < 0.01$, ***$p < 0.001$ (one-way ANOVA with Tukey's multiple comparisons test). **(D)** Representative results of WB for indicated proteins in HEK293 cells co-transfected with wild or Y104H SFTPC or No-TF. Signal intensities of BiP and CHOP in WB were quantified by densitometry, normalized by α-Tubulin, and indicated using a bar graph (n = 3). Data are presented as mean ± SD. *$p < 0.05$, **$p < 0.01$, ***$p < 0.001$ (one-way ANOVA with Tukey's multiple comparisons test).

Figure S2

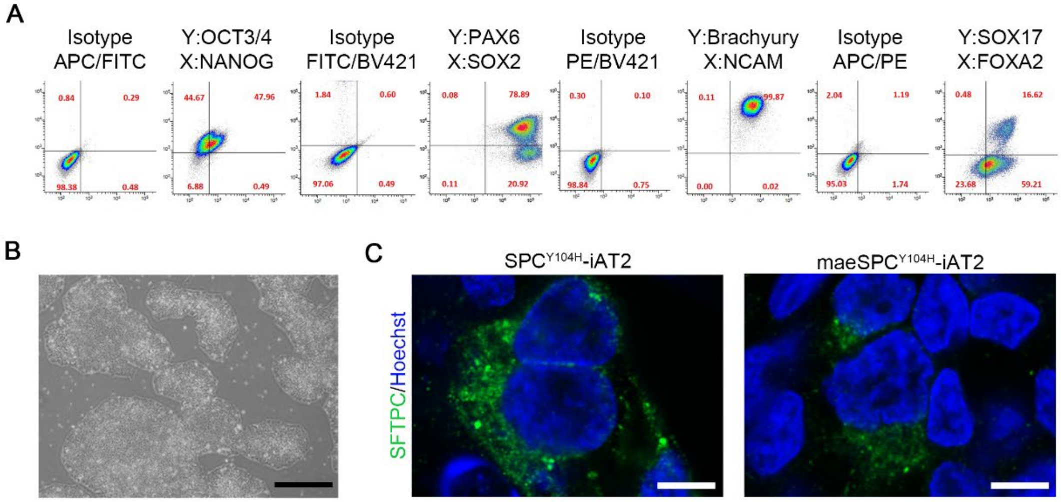

D Compounds that have passed screening using HEK293 cells (excluding CPT)

Compound No.	Compound Name	Known function
#562	Oridonin	Inhibitor of AKT1 and AKT2
#775	OSI-930	Inhibitor of C-Kit, KDR and CSF1R
#2352	Afatinib	Inhibitor of EGFR and HER2

Figure S2. Validation of patient-specific iPS cells, related to Figure 3.

(A) Flow cytometry for validating pluripotency. Stem cell marker expression of SPC^{Y104H}-iPSC was analyzed at the undifferentiated state. They were then differentiated into the tri-lineage germ layers. **(B)** Morphology of SPC^{Y104H}-iPSC. Scale bar = 100 μm. **(C)** Representative image of immunostaining for pro-SFTPC (green) and nuclear counterstaining with Hoechst 33342 (blue) in SPC^{Y104H}-iAT2 and maeSPC^{Y104H}-iAT2 cells. Scale bar = 5 μm. **(D)** A list of compounds that have passed screening using HEK293 cells (excluding CPT).

Figure S3

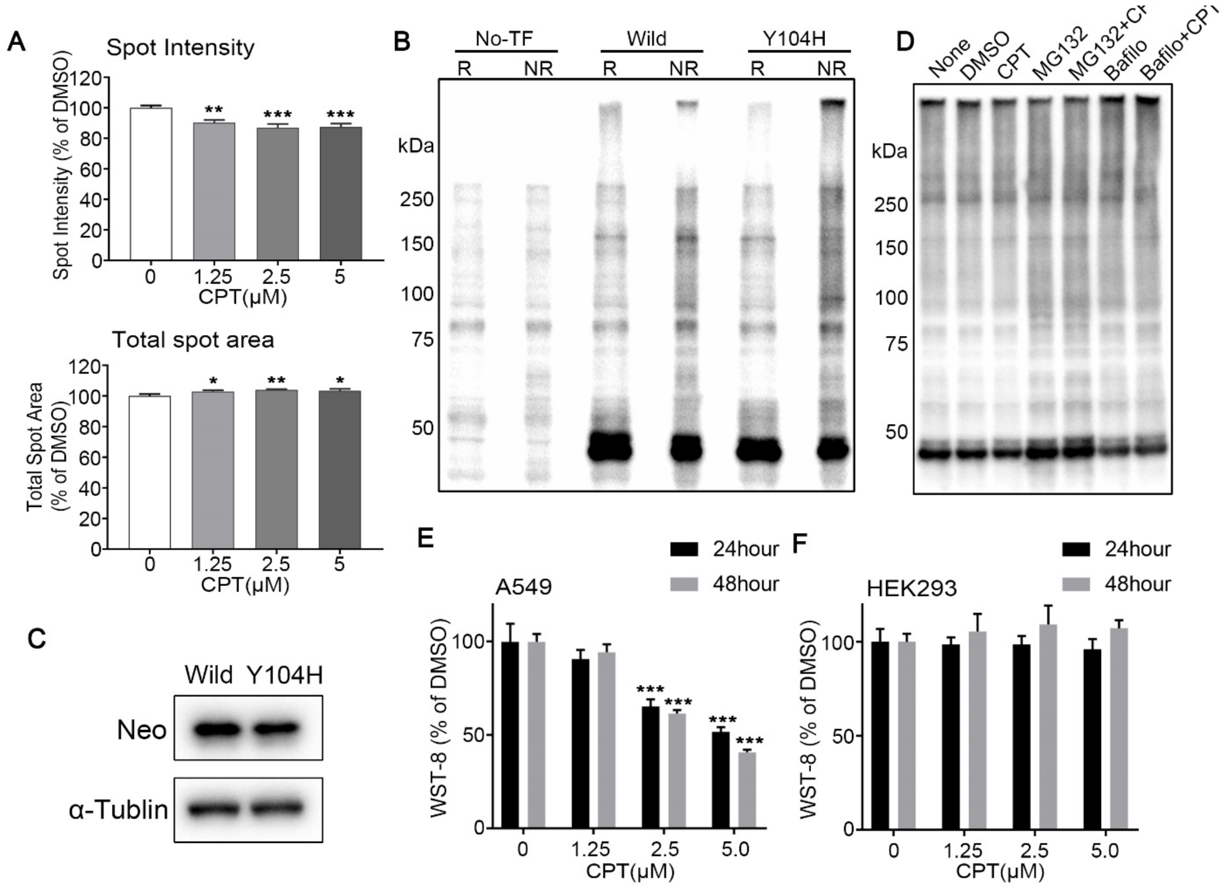

Figure S3. Assessment of the effects of CPT, related to Figure 4.

(A) Analysis of Spot Intensity and Total Spot Area of HEK293 cells transfected with the AcGFP-Y104H SFTPC for 4 h, followed by treatment with CPT (1.25, 2.5, or 5 µM) or DMSO (0.1%) for 48 h (n = 3, each sample is averaged over 81 fields of view). Data are presented as the mean ± SD. *$p < 0.05$, **$p < 0.01$, ***$p < 0.001$ (one-way ANOVA with Tukey's multiple comparisons test, compared with DMSO control). **(B)** Reduced (R) or non-reduced (NR) SDS-PAGE, followed by WB with anti-myc antibody for the lysates of A549 cells transfected with AcGFP-SFTPC (Wild or Y104H), or No-TF. Data are representative of three independent experiments. **(C)** WB with anti-Neo antibody for the lysates of A549 cells transfected with AcGFP-SFTPC (Wild or Y104H), the samples from Figure S3B. Data are representative of three independent experiments. **(D)** Non-reduced SDS-PAGE, followed by WB with anti-myc antibody for the lysates of A549 cells transfected with AcGFP-Y104H SFTPC for 4 h and subsequently treated with the indicated combination of 2.5 µM CPT, 5 nM Bafilomycin A1 (Bafilo), 5 µM MG132 or 0.1% DMSO for 24 h. Data are representative of three independent experiments. **(E)** The result of the WST-8 assay (% of DMSO) for A549 cells treated with CPT (1.25, 2.5, or 5 µM) or DMSO (0.1%) for 24 h or 48 h. (n = 3). Data are presented as the mean ± SD. ***$p < 0.001$ (one-way ANOVA with Tukey's

multiple comparisons test, compared with DMSO control). **(F)** The result of WST-8 assays (% of DMSO) for HEK293 cells treated with CPT (1.25, 2.5, or 5 μM) or DMSO (0.1%) for 24 h or 48 h. (n = 3). Data are presented as the mean ± SD. (one-way ANOVA with Tukey's multiple comparisons test, compared with DMSO controls).

Figure S4

A HALLMARK gene sets upregurated in SPC^{Y104H}-AEC

Gene set	NES	NOM *P* value	FDR *q* value
INTERFERON GAMMA RESPONSE	1.95	0.000	0.001
INTERFERON ALPHA RESPONSE	1.95	0.000	0.000
TNFA SIGNALING VIA NFKB	1.89	0.000	0.001
COMPLEMENT	1.79	0.000	0.004
EPITHELIAL MESENCHYMAL TRANSITION	1.75	0.000	0.005
INFLAMMATORY RESPONSE	1.70	0.001	0.008
COAGULATION	1.65	0.004	0.013
HYPOXIA	1.60	0.000	0.021
KRAS SIGNALING UP	1.51	0.004	0.042
UV RESPONSE DN	1.49	0.004	0.047
P53 PATHWAY	1.47	0.003	0.053

B Upregurated gene sets related to protein folding in SPC^{Y104H}-AEC

Gene set	NES	NOM *P* value	FDR *q* value
PROTEIN REFOLDING	2.02	0.000	0.037
CHAPERONE COFACTOR DEPENDENT PROTEIN REFOLDING	1.86	0.003	0.135
DE NOVO PROTEIN FOLDING	1.83	0.001	0.167
ATP DEPENDENT PROTEIN FOLDING CHAPERONE	1.80	0.001	0.169

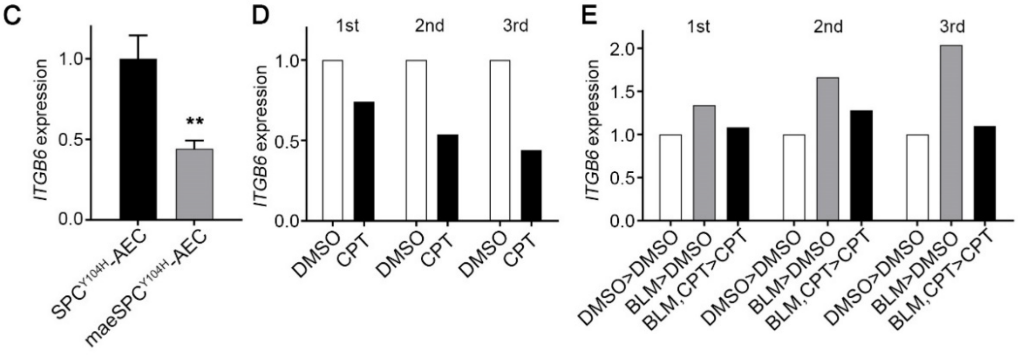

Figure S4. Characterization of alveolar organoids derived from SPC^{Y104H}-iPSC and the efficacy of CPT, related to Figure 5.

(A) List of significantly upregulated gene sets identified by GSEA analysis using Hallmark gene sets in SPC^{Y104H}-AEC. Gene sets (FDR q < 0.25, p < 0.01) are listed. **(B)** List of significantly upregulated protein folding-related gene sets identified by GSEA using C5: ontology gene sets. NES, normalized enrichment score; NOM, nominal; FDR, false discovery rate. **(C)** RT-qPCR of *ITGB6* in SPC^{Y104H}-AEC and maeSPC^{Y104H}-AEC (n = 3). Data are presented as mean ± SD. **p < 0.01 (Student's t-test). **(D)** RT-qPCR of *ITGB6* in FD-AOs derived from SPC^{Y104H}-iPSC treated with DMSO or 10 μM CPT from day 11 to day 17.. The results of the three independent experiments are shown, respectively. **(E)** RT-qPCR of *ITGB6* in SPC^{Y104H}-AEC treated with BLM, BLM, and 10 μM CPT or DMSO from day 11 to day 14, and 10 μM CPT or DMSO from day 14 to day 17. The results of the three independent experiments are shown, respectively.

Table S1. Predicted off-target site for CRISPR-sgRNA. Related to Figure 3.

chromosome		strand	start	end	sequence
chr1		+	231162428	231162448	CCTCGTGGTGC-TGA-TCCCAGC
chr2		+	188170622	188170643	CCTTGTGGTGAATGACT-CCAGC
chr3		+	154213532	154213554	CCTAGTGGTGGCATGACT-CCAGC
chr4		+	31026820	31026841	CCTAATGGTGCATGACTACCA-C
chr12		+	74514273	74514292	CCTC-TGGT-CATG-CTACCAGC
chr16		+	32919276	32919297	CCTCGTGGA-CATGACTACCTGC
chr16		+	55665425	55665445	GCTC-TGGTGCATGAC-ACCAGC
chr16_KI270728v1_random		+	1183261	1183282	CCTCGTGGA-CATGACTACCTGC
chr17		+	2056855	2056877	CCTGGTGGTGCATGACAACCTGC
chr17		+	38471457	38471478	CCTCGTGGA-CATGACTACCTGC
chr17		+	77716269	77716291	TCTTGTGGTGCATGCCTACCAGC
chr17_KI270857v1_alt		+	2506764	2506785	CCTCGTGGA-CATGACTACCTGC
chrX		+	122571907	122571929	CCTGGTGGTGCATGGAC-ACCAGC
chrY		+	20904496	20904516	CCT-GTGGTGAATGACT-CCAGC
chr3_KI270777v1_alt		-	68489	68511	GCTGG-AGTCATGCCACCACTAGG
chr6		-	1269207	1269228	GCTGGTAGT-ATGCGCCAAGAGG
chr8		-	117897292	117897313	GCTGGTAGTCATGCATCA-GATG
chr10		-	8411339	8411358	G-TGGTAG-CATGCACCAC-AGG
chr14		-	50053492	50053513	GCTGGCA-TCATGGACCACGAGG
chr16		-	33934574	33934595	GCAGGTAGTCATGT-CCACGAGG
chr19		-	15124853	15124873	GCTGGAAG-CA-GCACCACGAGG
chr22		-	23420541	23420561	GCTGGTGGTCATGCA-CA-GAGG
chr22_KI270878v1_alt		-	66499	66519	GCTGGTGGTCATGCA-CA-GAGG
chrX		-	76110558	76110579	GCTGGTAG-CATTCACCATGAGG

SUPPLEMENTAL REFERENCES

S1. Milara, J., Ballester, B., Safont, M.J., Artigues, E., Escriva, J., Morcillo, E., and Cortijo, J. (2021). MUC4 is overexpressed in idiopathic pulmonary fibrosis and collaborates with transforming growth factor beta inducing fibrotic responses. Mucosal Immunol *14*, 377-388. 10.1038/s41385-020-00343-w.

S2. Hancock, L.A., Hennessy, C.E., Solomon, G.M., Dobrinskikh, E., Estrella, A., Hara, N., Hill, D.B., Kissner, W.J., Markovetz, M.R., Grove Villalon, D.E., et al. (2018). Muc5b overexpression causes mucociliary dysfunction and enhances lung fibrosis in mice. Nat Commun *9*, 5363. 10.1038/s41467-018-07768-9.

S3. Higo, H., Ohashi, K., Tomida, S., Okawa, S., Yamamoto, H., Sugimoto, S., Senoo, S., Makimoto, G., Ninomiya, K., Nakasuka, T., et al. (2022). Identification of targetable kinases in idiopathic pulmonary fibrosis. Respir Res *23*, 20. 10.1186/s12931-022-01940-y.

S4. Suezawa, T., Kanagaki, S., Moriguchi, K., Masui, A., Nakao, K., Toyomoto, M., Tamai, K., Mikawa, R., Hirai, T., Murakami, K., et al. (2021). Disease modeling of pulmonary fibrosis using human pluripotent stem cell-derived alveolar organoids. Stem Cell Reports *16*, 2973-2987. 10.1016/j.stemcr.2021.10.015.

S5. Schneider, D.J., Wu, M., Le, T.T., Cho, S.H., Brenner, M.B., Blackburn, M.R., and Agarwal, S.K. (2012). Cadherin-11 contributes to pulmonary fibrosis: potential role in TGF-beta production and epithelial to mesenchymal transition. FASEB J *26*, 503-512. 10.1096/fj.11-186098.

S6. Ma, Z., Ma, C., Zhang, Q., Bai, Y., Mu, K., Liu, X., and Yang, Q. (2021). Role of CXCL16 in BLM-induced epithelial-mesenchymal transition in human A549 cells. Respir Res *22*, 42. 10.1186/s12931-021-01646-7.

S7. Li, Q., Deng, M.S., Wang, R.T., Luo, H., Luo, Y.Y., Zhang, D.D., Chen, K.J., Cao, X.F., Yang, G.M., Zhao, T.M., et al. (2023). PD-L1 upregulation promotes drug-induced pulmonary fibrosis by inhibiting vimentin degradation. Pharmacol Res *187*, 106636. 10.1016/j.phrs.2022.106636.

S8. Ptasinski, V., Monkley, S.J., Ost, K., Tammia, M., Alsafadi, H.N., Overed-Sayer, C., Hazon, P., Wagner, D.E., and Murray, L.A. (2023). Modeling fibrotic alveolar transitional cells with pluripotent stem cell-derived alveolar organoids. Life Sci Alliance *6*. 10.26508/lsa.202201853.
